# The production of viral vectors designed to express large and difficult to express transgenes within neurons

**DOI:** 10.1186/s13041-015-0100-7

**Published:** 2015-02-24

**Authors:** Roopashri Holehonnur, Srihari K Lella, Anthony Ho, Jonathan A Luong, Jonathan E Ploski

**Affiliations:** School of Behavioral and Brain Sciences and the Department of Molecular & Cell Biology, University of Texas at Dallas, 800 West Campbell Road, Richardson, TX 75080 USA

**Keywords:** NMDA, GluN2, AAV, Lenti, Viral vector, Amygdala, TRE3G

## Abstract

**Background:**

Viral vectors are frequently used to deliver and direct expression of transgenes in a spatially and temporally restricted manner within the nervous system of numerous model organisms. Despite the common use of viral vectors to direct ectopic expression of transgenes within the nervous system, creating high titer viral vectors that are capable of expressing very large transgenes or difficult to express transgenes imposes unique challenges. Here we describe the development of adeno-associated viruses (AAV) and lentiviruses designed to express the large and difficult to express GluN2A or GluN2B subunits of the N-methyl-D-aspartate receptor (NMDA) receptor, specifically within neurons.

**Results:**

We created a number of custom designed AAV and lentiviral vectors that were optimized for large transgenes, by minimizing DNA sequences that were not essential, utilizing short promoter sequences of 8 widely used promoters (RSV, EFS, TRE3G, 0.4αCaMKII, 1.3αCaMKII, 0.5Synapsin, 1.1Synapsin and CMV) and utilizing a very short (~75 bps) 3′ untranslated sequence. Not surprisingly these promoters differed in their ability to express the GluN2 subunits, however surprisingly we found that the neuron specific synapsin and αCaMKII, promoters were incapable of conferring detectable expression of full length GluN2 subunits and detectable expression could only be achieved from these promoters if the transgene included an intron or if the GluN2 subunit transgenes were truncated to only include the coding regions of the GluN2 transmembrane domains.

**Conclusions:**

We determined that viral packaging limit, transgene promoter and the presence of an intron within the transgene were all important factors that contributed to being able to successfully develop viral vectors designed to deliver and express GluN2 transgenes in a neuron specific manner. Because these vectors have been optimized to accommodate large open reading frames and in some cases contain an intron to facilitate expression of difficult to express transgenes, these viral vectors likely could be useful for delivering and expressing many large or difficult to express transgenes in a neuron specific manner.

**Electronic supplementary material:**

The online version of this article (doi:10.1186/s13041-015-0100-7) contains supplementary material, which is available to authorized users.

## Background

Viral vectors have become a common tool in neuroscience to express transgenes in a spatially and temporally restricted manner within the nervous system of numerous model organisms [1-5]. Among the various viral vector systems that are currently available, the most commonly used systems for targeting the nervous system are adeno-associated viruses (AAV) and lentiviruses.

Adeno-associated viruses are single stranded DNA viruses. Numerous serotypes of AAV possess a natural tropism for neurons and when these serotypes of AAV are infused into the CNS, neurons are the predominant cell type transduced. This property of AAV makes it convenient for directing ectopic transgene expression to neurons and eliminates the need to use neuronal specific promoters (i.e. synapsin, αCaMKII) to confine transgene expression to neurons [6-8]. Alternatively, lentiviruses derived from human immunodeficiency virus type 1 (HIV-1), are single stranded RNA viruses that are commonly pseudotyped with the vesicular stomatitis virus G-protein (VSV-G) and the VSV-G capsid provides the virus the ability to transduce many cell types. However due to this naturally broad tropism, VSV-G pseudotyped lentiviruses transduce glia cells very efficiently [9-12]. Therefore if neuronal specific gene expression is desired, then transgenes within VSV-G pseudotyped viruses must be controlled by neuronal specific promoters.

Despite the ubiquitous use of viral vectors to direct ectopic expression of trangenes within the nervous system, creating high titer viral vectors that are capable of expressing very large transgenes or difficult to express transgenes imposes unique challenges and this is in part due to limitations in the maximum viral genomes sizes that can be packaged into functional viruses. For example, AAV has an optimal viral genome size limit of ~4.7 - 5 Kb, which includes the ~150 bp inverted terminal repeats (ITRs) present at each end of the viral genome (combined ITR length is ~300 bps). This limit of course restricts the amount of genetic information that can be carried by the virus and therefore restricts the size and/or number of genes that can be packaged into AAV. Withstanding these limitations, there is convincing evidence that viral genomes as large as ~5.2 Kb can be packaged into functional viruses and even larger genomes have been used to create viruses that can confer ectopic transgene expression. It is thought that genomes larger than ~5.2 kb become truncated to ~5.2 kb during viral packaging and recombination between truncated genomes in the host cell enables the reconstitution of a functional transgene [13]. Producing AAV with genomes that are larger than the optimal genome size limit generally leads to a reduction in viral titer and a reduction of transgene expression. For *in vitro* purposes, this may be tolerable since usually a larger volume of virus can easily be used [14]. However for *in vivo* purposes the amount of virus that can be infused is constrained significantly. For example infusions of virus into the brain of a rodent can usually be no larger than a few microliters. It currently remains unclear how practical it is to use AAV with larger genomes for *in vivo* use. Lentiviruses possess a higher packaging limit than does AAV (~10 kb), but lentiviruses also exhibit reduced titers as viral genome size increases [15].

We were interested in developing viruses that could deliver a transgene to brain cells that would express the GluN2A or GluN2B subunit of the NMDA receptor, specifically within neurons. NMDA receptors are ionotropic glutamate receptors that are thought to be involved in synaptic plasticity, and multiple forms of learning [16] and their dysregulation are thought to occur in numerous psychiatric disorders [17,18]. Studies indicate that NMDAR signaling can be greatly influenced by the GluN2A/GluN2B ratio and this is due to inherent differences in calcium influx and cellular signaling mediated by these subunits [17]. Notably a switch in the GluN2A/B ratio occurs during development [19-21], in the aging brain [22-25], following synaptic activity [26-29] and irregularities of this ratio occur in models of psychiatric disease [30]. Therefore it would be of value if specific GluN2 subunits could be over expressed in models of learning and psychiatric disease, especially within specific circuits of the brain or at specific time points during development or before or after behavioral training to alter the GluN2A/B ratio. One way to accomplish this is by using viral vectors that could deliver these transgenes to neurons within the brain. However, the GluN2A and GluN2B coding regions are 4392 and 4446 bps respectively. Their large size creates challenges for creating a viral vector that can be produced at high titers and capable of conferring the expression of these transgenes efficiently *in vivo* in a neuron specific manner. Therefore to accomplish this goal, we systematically created a number of custom designed AAV and lentiviral vectors that were optimized for large transgenes, by minimizing DNA sequences that were not essential, utilizing short promoter sequences of 8 widely used promoters RSV, EFS, TRE3G, 0.4αCaMKII, 1.3αCaMKII, 0.5Synapsin, 1.1Synapsin and CMV, and utilizing a very short (~75 bps) 3′ untranslated sequence containing an SV40 based poly adenylation signal. Not surprisingly these promoters differed in their ability to confer expression of the GluN2 subunits, however surprisingly we found that the synapsin and αCaMKII, neuron specific promoters were incapable of conferring detectable expression of full length GluN2 subunits and detectable expression could only be achieved if the transgene included an intron or if the GluN2 subunit transgenes were truncated to only include the coding regions of their transmembrane domains. Collectively this study determined that the GluN2 subunits are difficult to express and that viral packaging limit and transgene promoter choice were critical factors for developing viral vectors designed to express GluN2 subunit transgenes. We suspect that many of the custom viral vectors we created could be of use by other investigators since they are optimized for larger transgenes. Additionally we created a lentiviral vector designed to express difficult to express transgenes specifically within neurons – this vector contains an intron downstream of a 1.3αCaMKII promoter. Here we describe our findings.

## Methods

### Plasmid creation

All viral vector plasmids were created using standard recombinant cloning procedures. A detailed description of how each viral plasmid was created for this study is described in Additional file 1. Viral genome sizes, and DNA oligonucleotides used for cloning purposes are provided in Additional file 2. Promoter DNA sequences are provided in Additional file 3. Additional relevant DNA sequences are provided in Additional file 4. DNA vector sequences are provided for all viral vectors created for this study in Additional file 5. AAV2 genome vectors plasmids, pAAV-Basic (*Vector Biolabs*) and pAAV (previously described, generous gift from Ralph DiLeone [31] were used for this study. The lentiviral plasmid pLenti7.3 DEST (*Invitrogen*) was modified and used for all lentiviral vectors for this study. The Flag-tagged rat GluN2A(pRK5) and mouse GluN2B(pRK5) containing plasmids were generously provided by Katherine Roche [32]. The 0.4αCamKII promoter was PCR amplified from rat genomic DNA. The mouse 1.3αCamKII promoter was PCR amplified from pAAV-CaMKII-RFP (Addgene # 22908) [33]. The rat synapsin promoters were PCR amplified from the L26 plasmid generously provided by Pavel Osten [12]. The RSV and CMV promoters were PCR amplified from pLenti7.3 DEST (*Invitrogen*). The TRE3G and EFS promoters were PCR amplified from Addgene vectors #(27569) and #(49535) [34] respectively. The AAV plasmids were propagated at 37°C in One shot Top Ten competent cells (*Invitrogen*). The lentiviral plasmids were propagated at room temperature in Stbl2 competent cells (*Life Technologies*). DNA plasmids were verified by DNA sequencing to be correct. Viral vector plasmids will be provided upon request and many of these will be made available through Addgene.

### Cell culture

293FT (*Invitrogen*) and Neuro2A (N2A) (ATCC, Cat #CCL-131) cells were grown in Dulbecco’s Modified Eagle Medium supplemented with 5% Glucose, 5 mM sodium pyruvate and 10% Fetal Bovine Serum *(Life Technologies)* in 5% CO_2_ humidified cell culture incubator.

### Immunocytochemistry

293FT and N2A cells were seeded at 65% confluency on pre-coated poly-l-lysine (0.1 mg/mL, *Sigma*) coverslips in 24-well plates. On the day of transfection, 0.8 ug of plasmid DNA was transfected into each well using the standard lipofectamine protocol (*Life technologies*). Plasmids containing EFS, RSV, TRE3G, CMV promoters were transfected into 293FT cells and plasmids expressing αCaMKII and synapsin promoters were transfected into N2A cells. After 24 hours, the coverslips were fixed using 4% paraformaldehyde in 1XPBS (pH7.4) (*Fisher Scientific*) for 20 minutes followed by a wash with 1X PBS(pH7.4) and then incubated with 0.2% Triton-X in 1XPBS (pH7.4) (*American Bioanalytical*) for 10 minutes on ice. The coverslips were next blocked in blocking serum (5% BSA (*Sigma*), 5% Goat serum (*Sigma*) in 1XPBS (pH7.4) for 90 minutes at room temperature. Following this, coverslips were incubated in 40 μls of mouse monoclonal anti-Flag primary antibody in blocking serum (1:200;F1804, *Sigma*) for 2 hours at 37°C. After the primary antibody incubation, the coverslips were washed twice with 1XPBS (pH7.4) and incubated with 40 μls of anti-mouse Texas Red antibody in blocking serum (1:1000, *Life technologies*) for 1 hour at room temperature. The coverslips were then rinsed thrice with 1XPBS (pH7.4) followed by incubation with 500 μls DAPI in 1XPBS (pH7.4) (1:25,000, *Invitrogen*) for 10 minutes at room temperature. The coverslips were rinsed three more times with 1XPBS (pH7.4) before mounting on superfrost slides (*Fisherbrand*) using Fluoromount-G (*Southern Biotech*). All images were visualized using 200X magnification using an Olympus BX51 upright fluorescence microscope with an Olympus DP71 Digital Camera and DP manager software. ICCs were performed in duplicates and the results were reproduced in at least 2 separate experiments. In order to compare the strength of promoters in AAV plasmid vectors, pRK5-GluN2A (positive control), AAV-CMV/TRE3G/EFS/RSV-GluN2A vectors, pTet-Off vector (*Clontech)* and AAV-CMV-GFP were transfected at 266.7 ngs each into one 24 well using the standard lipofectamine transfection protocol. The AAV-CMV-GFP plasmid was used to control for transfection efficiency. These transfections were performed in duplicate and were processed for ICC as mentioned above. Following this, images were acquired at 100X magnification at constant exposure time (Texas Red - 1.1 s; GFP – 66 ms) using an Olympus DP72 Digital Camera and Cellsens software (*Olympus*). At least 6 distinct fields of view were imaged and quantified for each group. Using Image-J software, the mean OD for Texas Red (GluN2A), GFP and DAPI were calculated. The GFP signal served as a transfection efficiency control and the DAPI signal served as a cell number control. No difference in GFP signal or DAPI signals were found among the groups indicated that the transfection efficiency and cell number did not vary significantly among these groups. To obtain the Texas Red (GluN2A) expression levels for each group, the Texas Red signal was normalized to the GFP signal and these data were plotted as average percent expression as compared to the control group. Error bars represent standard error of mean. Parametric statistics using one-way ANOVA with a Fisher PLSD post-hoc test was applied to evaluate statistical differences among AAV vector transgene expression. Differences were considered significant if, p < 0.05.

### AAV virus production/purification

An AAV2 genome plasmid harboring the gene of interest (Flag-GluN2A, Flag-GluN2B, GFP) was packaged into functional viruses that were pseudotyped with AAV DJ or AAV DJ8 capsid proteins to produce AAV2/DJ and AAV2/DJ8 viruses. Pseudotyped viruses were produced using a triple-transfection, helper-free method using 293FT cells and the resultant viruses were purified on an iodixanol step gradient, as previously described [31,35]. Purified viruses were titered using a qRT-PCR based method to determine the number of DNAse resistant viral particles and the results were reported as genomes copies (GC)/mL and this was performed as previously described [8] using PCR primers specific for GluN2A (*Life Technologies*, Cat#Rn00561341_m1), GluN2B (*Life Technologies*, Cat# Mm00433820_m1) and GFP (*Life technologies*, Cat# Mr04329676_mr).

### Lenti virus production/purification

Small scale lentiviruses were made following the Invitrogen Forward Transfection protocol. Briefly, the cells were seeded at 65% confluency in pre-coated poly-l-lysine (0.1 mg/mL, Sigma) 10 cm plates a day before transfection. On the day of transfection, 3 μg of the lentiviral genome plasmid containing the gene of interest and 9 ug of the lentiviral packaging plasmids: pLP1 (3.34 μg), pLP2 (2.21 μg) and pLP/VSV-G (3.34 μg) were transfected using the Virapower Lentivirus Forward Transfection (*Life Technologies*) protocol per 10 cm plate. At seventy-two hours post transfection, the supernatant was collected and centrifuged at 3000 g for 15 minutes and filtered through 0.45 μm PVDF filters (*Fisher brand*) to remove cell debris. Following this, the lentivirus was concentrated using PEG-it (*System Biosciences*). At Forty-eight hours following addition of PEG-it, the viral solution was centrifuged using a swinging bucket rotor at 1500 g for 30 minutes to pellet the virus and the viral pellet was resuspended in 100 μls of cold 1XPBS (pH7.4). The lentiviruses were then aliquoted in 10 μl volumes and frozen at −80°C. Large scale lentivirus production was performed as previously described [36,37]. Briefly, 3 (15 cm) plates pre-coated with poly-l-lysine (0.1 mg/mL, Sigma) were transfected with 27 ug of lentiviral genome plasmid containing the gene of interest and 84 ug of lentiviral packaging plasmids pLP1 (31.5 μg), pLP2 (21 μg) and pLP/VSV-G (31.5 μg) diluted in 10.5 ml of optimem and mixed with 240 μl of lipofectamine diluted in 10.5 ml optimem (5 minute incubation) and was further incubated for 20 minutes. Following this, existing media in each plate was replaced with fresh 13 ml of optimem (*Life Technologies*) and 7 ml of the transfection mixture was added. At twenty hours post transfection, the existing media from each plate was replaced with 22 ml of warm optimem media. Forty eight hours after transfection, the supernatant was collected, centrifuged for 1200 rpm for 15 minutes at 4°C followed by filtering through 0.45 μm PVDF filter (*Millipore*). The viral supernatant was then added into Beckmann ultracentrifuge tubes (344058) and spun at 26,000 rpm for 90 minutes at 4°C in a Beckmann SW 32-Ti swinging bucket rotor. Following ultracentrifugation, the supernatant was discarded carefully and the viral pellet in each tube was resuspended in 40 μl of cold 1XPBS. The viruses were then aliquoted into 10 μl aliquots and stored at −80°C. Purified lentiviruses were titered using an qRT-PCR based method (adapted from Global UltraRapid Lentiviral Titer Kit, *System Biosciences*) to determine the number of integrated lentiviral genomes directly from lysates of cells and the results were reported as genomes copies (GC)/mL. Briefly, 2 μl of concentrated lentivirus were added to 293FT cells grown in 24 well plates, in triplicate. Lentiviruses concentrated using ultracentrifugation appeared to transduce cells in culture poorly when compared to viruses concentrated using PEG-it reagent, therefore we used TranDux reagent (*System Biosciences*) to enhance *in vitro* transduction of ultracentrifugation concentrated lentiviruses. At seventy-two hours after addition of the viruses, the cells were harvested using 75 μl of lysis buffer (*System Biosciences*) and heated for 2 minutes at 95°C. The supernatant was then centrifuged at 14,000 rpm for 2 minutes and 5 μl of this solution was used per standard 20 μL Taqman PCR assay (*Applied Biosystems*). One μl of a 20X Taqman custom WPRE Primer/Probe for pLenti-WPRE (WPRE FP: CCGTTGTCAGGCAACGTG, WPRE RP: AGCTGACAGGTGGTGGCAAT, WPRE Probe: 5′FAM-TGCTGACGCAACCCCCACTGGT-TAMRA 3′) was used per reaction. Samples were prepared and loaded onto a 96 well plate in triplicate and quantitated using a CFX96 Real-time PCR system (*BioRad*) using the standard cycling parameters specified by Global UltraRapid Lentiviral Titer Kit (*System Biosciences*). Lentiviral genome copies were quantitated based on a standard curve prepared by serially diluting the pLenti-0.4αCaMKII-GFP plasmid in (10 mM Tris pH 7.4, 10 μg/ml of yeast tRNA solution) across eight, three fold serial dilutions ranging from 1 × 10^5^ to 1× 10^9^ copies of the viral genome plasmid per PCR reaction (as described above). Final viral titers were computed based on the standard curve and reported as genome copies GC/ml.

### AAV and lentivirus transduction *in vitro*

293FT cells grown in 24 well plates were each transduced with 0.8 - 1.3 μl of AAV containing ~1E + 13 GC/mL virus in 200 μl of L-DMEM (growth media with 2% FBS) media for 2 hours followed by addition of 400 μl of H-DMEM (growth media with 18% FBS) media. 293FT cells grown in 24 wells were transduced with 2, 10 or 20 μl of PEG-it purified lentivirus containing 1E + 9 GC/mL - 1E + 10 GC/mL virus diluted into 500 μl of DMEM growth media. The cells were incubated for 48 hours before being processed for ICCs as described above.

### Subjects

Adult male Sprague Dawley rats (*Harlan*) weighing 300 – 400 g and adult C57BL/6 αCaMKII-tTA mice (*Jackson Laboratories,* cat # 003010 B6) [38] weighing 20 – 25 g were housed individually and maintained on a 12 hour light/dark cycle. Food and water were provided *ad libitum* throughout the experiment. Animal use procedures were in accordance with the National Institutes of Health Guide for the Care and Use of laboratory animals and were approved by the University of Texas at Dallas.

### AAV and lentivirus transduction *in vivo*

Under a mixture of ketamine (100 mg/kg) and xylazine (10.0 mg/kg) anesthesia, rats and mice were stereotaxically implanted bilaterally with a 28 gauge infusion cannula targeting the basolateral amygdala (BLA) [rat: AP −2.9, ML ±5.2, DV −8.6, mice: AP −1.6 ML ±3.3 DV −4.95]. AAV and lentiviruses were infused essentially as described previously [8]. One microliter of AAV virus at ~1E + 13 GC/mL and lentivirus at 1E + 9 GC/mL - 1E + 11 GC/mL were bilaterally infused into the rat BLA over 15 minutes at a rate of 0.07 μl/min. Half of one microliter of lentiviruses ~1E + 11 GC/mL designed to express TRE3G-GFP and TRE3G-Flag-GluN2A/B were bilaterally infused into the BLA of αCaMKII-tTA mice over 15 minutes at the rate of 0.07 μl/min.

### Immunohistochemistry (IHC)

Twenty one days post infusion of AAV viruses and ten days post infusion of lentiviruses, the animals were anesthetized with an overdose of chloral hydrate (250 mg/kg) and then perfused with 1XPBS-A (1X phosphate buffer, 150 mM NaCl) and 10% buffered formalin *(Fisher scientific)* for animals infused with GFP viruses or with 1XPBS (pH = 7.4) *(American Bioanalytical)* and 4% paraformaldehyde in 1XPBS (pH7.4) for animals infused with GluN2 viruses. Following brain extraction, the brains were fixed in formalin/4% paraformaldehyde *(Fisher scientific)* in 1XPBS (pH7.4) for 3–4 hours, followed by cryoprotection in 30% sucrose 1XPBS-A (1X Phosphate buffer, 150 mM NaCl)/1X PBS pH7.4, for 3–4 days. Following cryoprotection, the brains were frozen and 40 μm coronal sections were obtained using a cryostat. For viruses designed to express GFP, images were obtained at 100X using an Olympus BX51 upright fluorescence microscope with an Olympus DP71 Digital Camera and DP manager software. For viruses designed to express Flag-GluN2A/GluN2B, the slices were processed for Flag-IHC. The slices were first rinsed with 1XPBS (pH7.4) for 10 min followed by incubation for 60 min with blocking buffer (1X PBS, 5% normal goat serum, 0.3% Triton X-100). Next the slices were incubated overnight at 4°C with Flag antibody (1:800; M2 8146, *Cell signaling*), diluted in antibody dilution buffer (1XPBS, 1% BSA, 0.3% Triton X-100). For secondary antibody staining, the slices were incubated for 2 hours at room temperature in a solution of 1:1000 dilution of anti-mouse Texas Red antibody (*Life Technologies)*. The slides were coverslipped with Vectashield Hard Set Mounting Medium (*Vector Laboratories*). IHC images were captured at 100X magnification using an Olympus BX51 upright fluorescence microscope with an Olympus DP71 Digital Camera and DP manager software. For Flag and NeuN co-localization IHC, the slices were processed with Flag and NeuN antibodies. Briefly, the slices were prepared and fixed as mentioned above. The slices were first rinsed with 1X-PBS (pH7.4) for 10 min followed by incubation for 60 min with blocking buffer (1X PBS, 2.5% normal goat serum, 2.5% normal donkey serum, 0.3% Triton X-100). Next the slices were incubated overnight at 4°C with an Anti-Flag antibody (1:500; PA1-984B, *Thermoscientific* and Anti-NeuN antibody (1:500; MAB377 Millipore, Billerica, MA) diluted in antibody dilution buffer (1XPBS, 1% BSA, 0.3% Triton X-100). For secondary antibody staining, 1:1000 dilution of anti-rabbit Texas Red antibody (*Life Technologies)* and 1:200 dilution of fluorescein isothiocyanate (FITC) conjugated anti-Mouse IgG (AP192F; Millipore) was used. The slices were incubated for 2 hours at room temperature. The slides were coverslipped with Vectashield Hard Set Mounting Medium (*Vector Laboratories*). IHC images were captured at 100X magnification using an Olympus BX51 upright fluorescence microscope with an Olympus DP71 Digital Camera and DP manager software under Tx-red and FITC channels. The number of transduced (Flag-staining) cells that were also NeuN positive was determined by quantifying the number of cells showing Flag-staining that also co-localized with the NeuN FITC signal within an area of 19008.78 μm2 per slice, (n = 3). Cells were manually quantified using Adobe Photoshop CS5 software and the co-localization results were reported as a percentage of total Flag-staining positive cells counted.

## Results

### Neuron specific promoters are incapable of conferring detectable expression of GluN2A/B, but they can confer expression of GluN2A/B C-terminal tail deletion mutants and GFP

**Figure 1:**
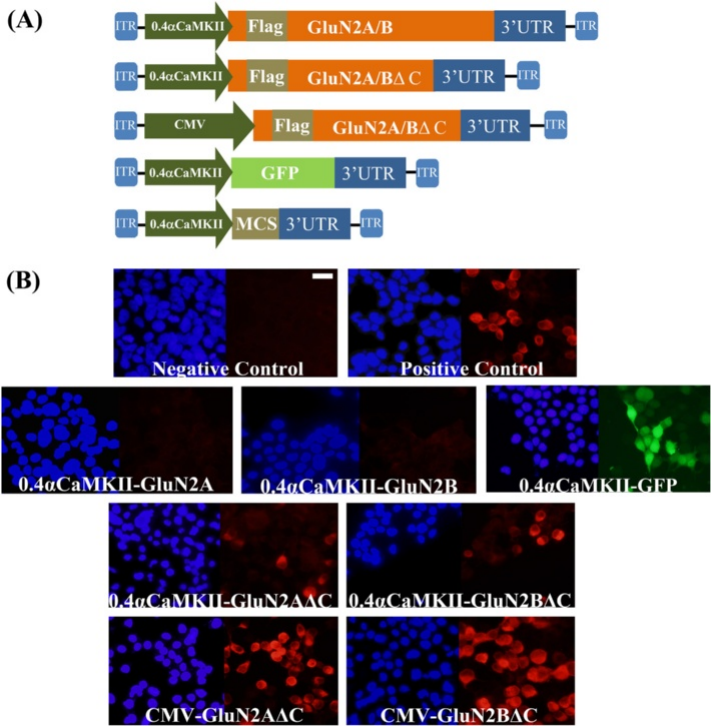
**Adeno-associated viral vector maps and GluN2 and GFP transgene expression from these AAV viral vector plasmids** ***in vitro*** **. (A)** Viral genome maps for pAAV-Basic vector backbones containing the following transgenes: Flag-GluN2A/B, Flag-GluN2A/B with C-terminal deletions (GluN2A/BΔC) and GFP. These transgenes are controlled by the 0.4αCaMKII promoter or CMV promoter as indicated. Each of these vectors contain a ~200 bps 3′UTR that contains an SV40 based poly-adenylation signal. GFP = green fluorescence protein, ITR = inverted terminal repeat, MCS = multiple cloning site **(B)** AAV plasmids designed to express, Flag-GluN2A/B, GFP, and Flag-GluN2A/BΔC from a 0.4αCaMKII promoter and Flag-GluN2A/BΔC from a CMV promoter were transfected into N2A cells. Twenty four hours after transfection, native GFP expression was observed via fluorescence microscopy and Flag-GluN2 expression was observed via ICC and fluorescence microscopy. Images depict DAPI stained nuclei with the same fields viewed for GFP or Flag-GluN2 (Texas Red) transgene expression. Flag-GluN2A/B did not exhibit detectable expression. However GFP and Flag-GluN2A/BΔC exhibited convincing expression. Untransfected cells and cells transfected with pRK5-Flag-GluN2 plasmids were processed as negative and positive ICC controls respectively. (Scale bar = 20 μm).

**Figure 2:**
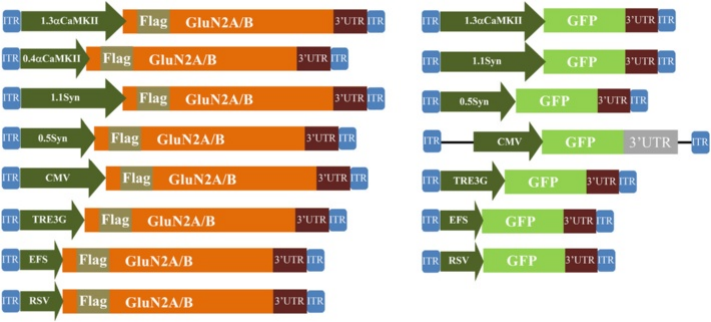
**Vector maps of AAV vectors optimized for large transgenes.** Viral genome maps for pAAV vector backbones containing either the Flag-GluN2A/B or GFP transgenes. Each of these vectors contain a ~75 bps 3′UTR that contains an SV40 based poly-adenylation signal. These transgenes are controlled by one of the following promoters as indicated: 1.3αCaMKII, 0.4αCaMKII, 1.1Synapsin, 0.5Synapsin, CMV, TRE3G, EFS, or RSV. The promoters are depicted as their approximate relative sizes to one another. GFP = green fluorescence protein, ITR = inverted terminal repeat, MCS = multiple cloning site.

**Figure 3:**
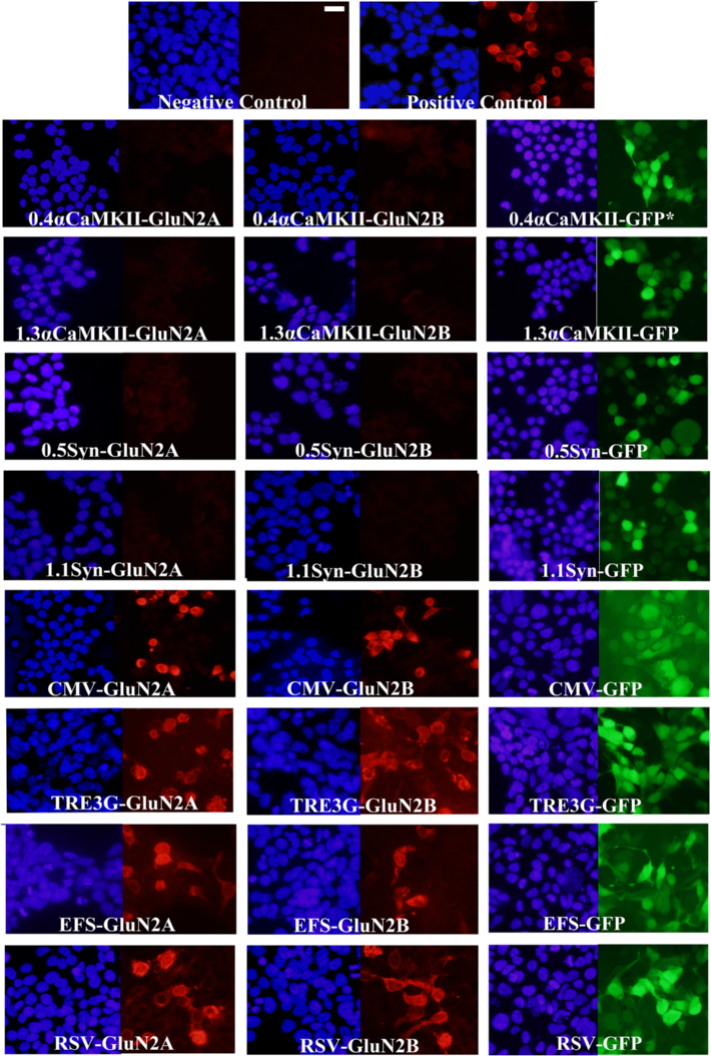
**GluN2 and GFP transgene expression** ***in vitro*** **from optimized AAV vector plasmids.** Representative ICC images from transfections with optimized AAV plasmids designed to express Flag-GluN2A/B or GFP transgenes utilizing one of the following promoters: 1.3αCaMKII, 0.4αCaMKII, 1.1Synapsin, 0.5Synapsin, CMV, TRE3G, EFS, or RSV. Plasmids containing transgenes controlled by the neuron specific, 0.4αCaMKII, 1.3αCaMKII, 0.5synapsin and 1.1synapsin promoters were transfected into N2A cells. Plasmids containing transgenes controlled by CMV, TRE3G, EFS and RSV promoters were transfected into 293FT cells. TRE3G promoter containing plasmids were co-transfected with the pTet-Off plasmid. Twenty four hours after transfection, native GFP expression was observed via fluorescence microscopy and Flag-GluN2 expression was observed via ICC and fluorescence microscopy. Images depict DAPI stained nuclei with the same fields viewed for GFP or Flag-GluN2 (Texas Red) transgene expression. Plasmids with neuron specific promoters were able to confer GFP expression but were not able to confer GluN2 expression. Plasmids containing transgenes controlled by CMV, TRE3G, EFS and RSV promoters were capable of conferring GluN2 and GFP expression. Untransfected cells and cells transfected with pRK5-Flag-GluN2 plasmids were processed as negative and positive ICC controls respectively. (Scale bar = 20 μm).

**Figure 4:**
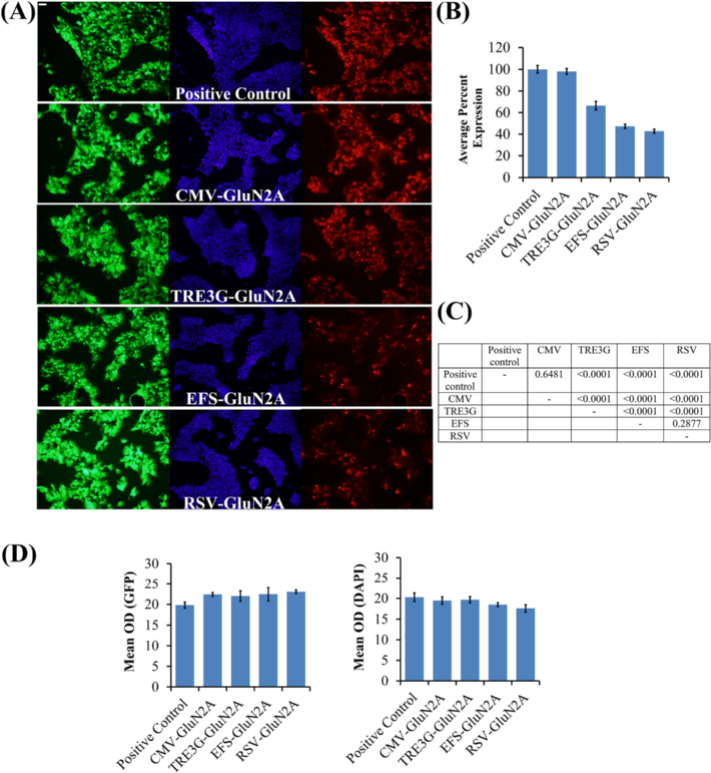
**The CMV, TRE3G, EFS and RSV promoters differ in their ability to confer GluN2A expression** ***in vitro*** **. (A)** Representative ICC images for Flag-GluN2A expression and associated GFP and DAPI staining from transfected cells. In this experiment, AAV plasmids designed to express GluN2A from one of the following promoters, (CMV, TRE3G, EFS, RSV), were cotransfected into 293FT cells with a plasmid containing a CMV-GFP transgene and the pTet-Off plasmid. Twenty four hours after transfection, native GFP expression was observed via fluorescence microscopy and Flag-GluN2 expression was observed via ICC and fluorescence microscopy. Images depict DAPI stained nuclei with the same fields viewed for GFP and Flag-GluN2 (Texas Red) transgene expression. GFP was used as a transfection efficiency control. **(B)** Quantitation of Flag-GluN2A expression data presented in **(A)**. The Texas Red (Flag-GluN2A) expression levels were normalized to GFP expression levels and these data were plotted as average percent expression as compared to the control group. n = 6, Error bars represent standard error of the mean (SEM). The AAV-CMV-GluN2A plasmid conferred similar Flag-GluN2A expression as the positive control (pRK5-Flag-GluN2A) and these exhibited higher levels of Flag-GluN2A expression, as compared to TRE3G, EFS, and RSV GluN2A containing plasmids. **(C)** *p* values for one-way ANOVA with Fisher’s PLSD post-hoc test for **(B)**. Differences were considered significant if, *p* < 0.05. **(D)** Mean OD for GFP expression and DAPI staining for Flag-GluN2A expression data presented in **(A)**. One-way ANOVA revealed that GFP levels (F(4,25) = 1.532; p = 0.2235) and DAPI levels (F(4,25 = 1.652; p = 0.1925), did not differ significantly indicating that transfection efficiency did not differ among the groups and the number of cells quantified did not differ significantly among the groups.

We were interested in developing viruses that could deliver GluN2A or GluN2B subunit transgenes into brain cells and express these transgenes specifically within neurons. Here we describe developing AAV for this purpose. Because AAV has an optimal packaging limit of ~4.7 - 5.0 kb we attempted to minimize the transgene sizes as much as possible. The first viral vectors created were done so using a commercially available AAV2 genome plasmid (pAAV-Basic, Vector Biolabs) and these vectors were designed to express the following transgenes: Flag-GluN2A, Flag-GluN2B, Green fluorescent protein (GFP), C-terminal deletion mutant of Flag-GluN2A (Flag-GluN2AΔC) and a C-terminal deletion mutant of Flag-GluN2B (Flag-GluN2BΔC). These transgenes were designed with the short version of the αCaMKII promoter (0.4), and ~200 bps 3′ UTR that contained an SV40 based polyadenylation signal sequence. Versions of the Flag-GluN2AΔC and Flag-GluN2BΔC plasmids were also created that contained a cytomegalovirus (CMV) promoter instead of the 0.4αCaMKII promoter. These plasmids were transfected into Neuro 2A(N2A) cells and 24 hours later anti-Flag immunocytochemistry (ICC) was performed to detect the ectopic expression of the Flag-tagged GluN2 transgenes and the native GFP fluorescence for the GFP transgene was detected using standard fluorescence microscopy. Transgene expression was detected for 0.4αCaMKII-GFP, 0.4αCaMKII-Flag-GluN2AΔC, 0.4αCaMKII-Flag-GluN2BΔC, CMV-Flag-GluN2AΔC, and CMV-Flag-GluN2BΔC plasmids. In contrast we were not able to detect expression of 0.4αCaMKII-Flag-GluN2A and 0.4αCaMKII-Flag-GluN2B. Since each of the 0.4αCaMKII promoter containing plasmids were essentially identical by design, these data indicated that 0.4αCaMKII promoter could confer gene expression within this cell line, but the cytoplasmic C-terminal domains of GluN2A and GluN2B must interfere with the expression of these transgenes or the stability of GluN2 gene products (Figure 1). Each of the viral vector plasmids produced using pAAV-Basic will be denoted with an asterisk(*) to differentiate them from other vectors. Next, new viral vector plasmids were generated using a different AAV2 genome plasmid ([31], see Methods), because it allowed the transgenes to be ligated directly adjacent to the ITRs, which eliminated 145 bps of unnecessary DNA compared to the pAAV-Basic vector. In these newer versions of viral vectors, a shorter ~75 bps 3′UTR was used, which contained an SV40 based polyadenylation signal sequence – this further increased the available space that could be used for potential transgene DNA, effectively increasing the size of transgenes that could be delivered by AAV (Figure 2). The Flag-GluN2A, Flag-GluN2B, and GFP coding regions were cloned into these vectors and subsequently versions of these plasmids were generated to contain one of the following different promoters: 0.4αCaMKII, 1.3αCaMKII, 0.5Synapsin, 1.1Synapsin, Cytomegalovirus (CMV), Short form of Elongation Factor-1α (EFS), Rous Sarcoma Virus (RSV) and third generation Tet-inducible promoter (TRE3G). The plasmids with neuron specific promoters (i.e. CaMKII, Synapsin) were transfected into N2A cells, and the other plasmids were transfected into 293FT cells. The plasmids containing the TRE3G promoter were cotransfected with the pTet-Off plasmid (*Clontech*), containing the tetracycline transactivator (tTA) transgene, which is necessary to confer expression from the TRE3G promoter. Transgene expression from each of the AAV plasmids was examined 24 hours post transfection. GFP was expressed robustly by each promoter, but GluN2A and GluN2B were only expressed by the CMV, EFS, RSV, and TRE3G promoters (Figure 3). Notably none of the plasmids with neuron specific promoters (i.e. synapsin, CaMKII) were capable of conferring detectable expression of the full length Flag GluN2 transgenes and the CMV, TRE3G, EFS, and RSV promoters exhibited a differential ability to drive detectable expression of the GluN2 transgenes, where the CMV and TRE3G promoters induced the best expression and the RSV and EFS promoters comparatively induced less expression (Figure 4). Because these vectors have been optimized to minimize unnecessary DNA sequences, and to contain short promoters and 3′UTR sequences, these vectors likely could be useful for the expression of many large transgenes. GFP or GluN2 coding regions can be excised from these vectors and other transgenes can easily be inserted into these vectors.

### AAV was not capable of conferring full length GluN2A/B transgene expression *in vitro* or *in vivo*

**Figure 5:**
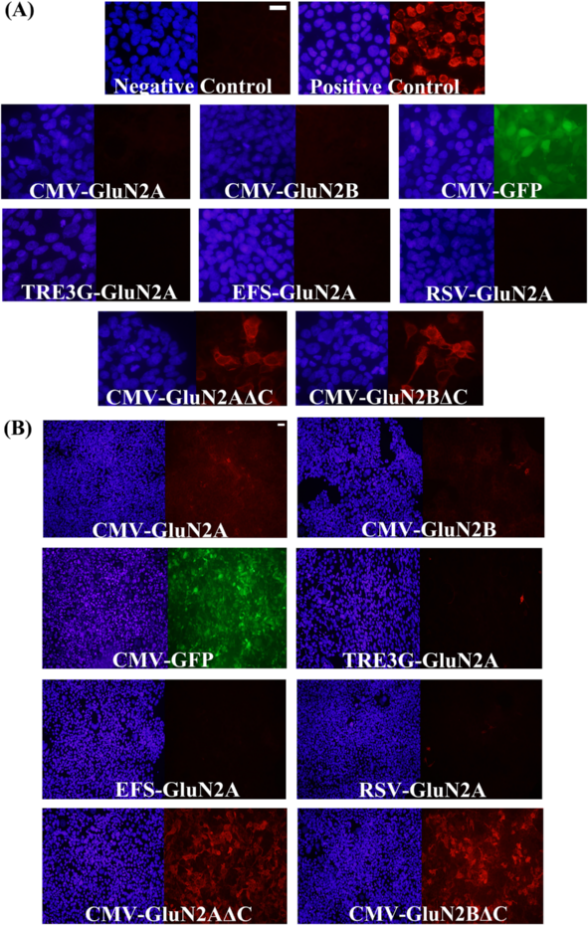
**GluN2 and GFP transgene expression** ***in vitro*** **mediated by adeno-associated virus. (A)** In this experiment, 293FT cells were transduced with AAV viruses designed to express GFP from a CMV promoter, Flag-GluN2A from the CMV, TRE3G, EFS, or RSV promoters, and Flag-GluN2B and GluN2A/BΔC from a CMV promoter. TRE3G promoter containing viruses were transduced into cells that were also transfected with the pTet-Off plasmid. Forty eight hours after viral transduction, native GFP expression was observed via fluorescence microscopy and Flag-GluN2 expression was observed via ICC and fluorescence microscopy. Images depict DAPI stained nuclei with the same fields viewed for GFP or Flag-GluN2 (Texas Red) transgene expression. Non-transduced cells and cells transfected with pRK5-Flag-GluN2 plasmids were processed as negative and positive ICC controls respectively. AAV designed to express Flag-GluN2 from the CMV, TRE3G, EFS, or RSV promoters did not confer convincing transgene expression. However AAV designed to express GFP or GluN2A/BΔC exhibited expression in > 90% of cells. (Scale bar = 20 μm) **(B)** Depicts similar images as depicted in A, but at lower magnification. (Scale bar = 50 μm).

Next we wanted to determine if the AAV viral plasmids that could confer gene expression of the GluN2 subunits could be packaged into functional virus and could be used to transduce cells grown in culture (i.e. *in vitro*) to induce ectopic transgene expression. AAV viruses pseudotyped as AAV2/DJ were produced using the following viral vector plasmids: EFS-GluN2A, RSV-GluN2A, TRE3G-GluN2A, CMV-GluN2A, CMV-GluN2B, CMV-GluN2AΔC, CMV-GluN2BΔC, and CMV-GFP. 293FT cells were treated with equal amounts of virus and examined for transgene expression 48 hours following application of the virus. Cells that were transduced with the TRE3G-GluN2A virus were transfected with the pTet-Off plasmid 6 hours before the virus was applied to the cells. We observed that the CMV-GFP virus which has a viral genome size of 3045 bps exhibited expression in >90% of cells. Similarly CMV-GluN2AΔC and CMV-GluN2BΔC viruses which have viral genomes sizes of 3893 bps and 3879 bps respectively, also exhibited expression in >90% of cells indicating that these viral genomes were successfully packaged into functional viral particles (Figure 5). However, AAV-CMV-GluN2A, AAV-CMV-GluN2B, AAV-TRE3G-GluN2A, AAV-EFS-GluN2A, and AAV-RSV-GluN2A, which have the viral genome sizes of, 5411, 5476, 5228, 5077, and 5055 bps respectively, demonstrated very little expression of the GluN2 transgene. Expression of the GluN2 transgene was observed in only 15–35 cells for the entire coverslip. We suspect that the lack of expression observed from AAV-CMV-GluN2A, AAV-CMV-GluN2B, AAV-TRE3G-GluN2A is likely due to the fact that these viruses were produced with viral genomes that were over the optimal packaging limit and we suspect that AAV-EFS-GluN2A, and AAV-RSV-GluN2A may not have been capable of conferring GluN2 transgene expression because these viruses were also produced with genomes above the optimal size and that the RSV and EFS promoters are not capable of conferring robust expression of the GluN2A transgene, as compared to the CMV and TRE3G promoters. AAV viruses that contain neuron specific promoters were not examined *in vitro*, because N2A cells are not transduced well by AAVDJ. We have examined numerous serotypes of AAV for their ability to transduce N2A cells but we have not been able to find one (data not shown).

**Figure 6:**
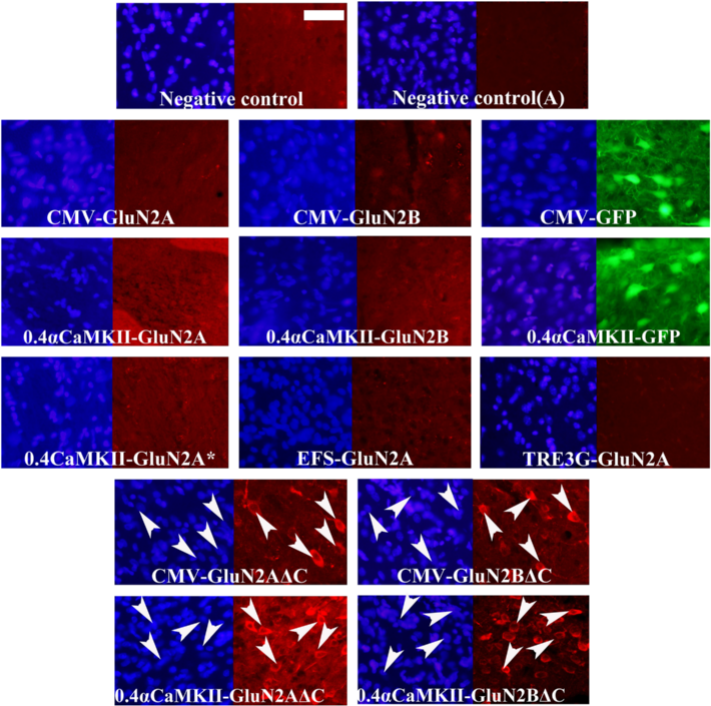
**GluN2 and GFP transgene expression** ***in vivo*** **mediated by adeno-associated virus.** In this experiment, AAV viruses designed to express Flag-GluN2A/B, GFP, and Flag-GluN2A/BΔC from a CMV, EFS, TRE3G or 0.4αCaMKII promoter, as indicated, were infused into the basolateral complex of the amygdala (BLA). All viruses were infused into the rat BLA, except for the TRE3G promoter containing viruses – these viruses were infused into the BLA of αCaMKII-tTA transgenic mice. Twenty one days following viral infusion, coronal sections were prepared that contained the BLA and native GFP expression was observed via fluorescence microscopy and Flag-GluN2 expression was observed via immunohistochemistry(IHC) and fluorescence microscopy. Images depict DAPI stained nuclei with the same fields viewed for GFP or Flag-GluN2 (Texas Red) transgene expression. Viruses designed to express GFP and Flag-GluN2A/BΔC exhibited convincing and robust transgene expression *in vivo*. Viruses designed to express Flag-GluN2A/B were not capable of conferring GluN2 expression *in vivo*. *indicates virus derived from pAAV-Basic vector. Coronal sections from naïve controls were processed as negative controls for Flag-IHC and coronal sections from wild type mice were processed as controls (Control A) for mice infused with AAV-TRE3G-Flag-GluN2A.

Along with testing many of these viruses *in vitro*, we also infused the following viruses *in vivo*, into the basolateral amygdala: AAV-0.4αCaMKII-GluN2A*, AAV-0.4αCaMKII-GluN2A, AAV-0.4αCaMKII-GluN2B, AAV-CMV-GluN2A, AAV-CMV-GluN2B, AAV-EFS-GluN2A, AAV-CMV-GluN2AΔC, AAV-CMV-GluN2BΔC, AAV-0.4αCaMKII-GluN2AΔC, AAV-0.4αCaMKII-GluN2BΔC, AAV-TRE3G-GluN2A, AAV-CMV-GFP, and AAV-0.4αCaMKII-GFP. Twenty one days following viral infusion, the animals were sacrificed, coronal brains sections were prepared that contained the amygdala and the tissue was processed for anti-Flag IHC or visualization of native GFP fluorescence as appropriate. All viruses were infused into rats, except the viruses containing the TRE3G promoter. These TRE3G containing viruses were infused into αCaMKII-tTA mice [38]. AAV-CMV-GluN2AΔC, AAV-CMV-GluN2BΔC, AAV-0.4αCaMKII-GluN2AΔC, AAV-0.4αCaMKII-GluN2BΔC, AAV-CMV-GFP, and AAV-0.4αCaMKII-GFP were all capable of conferring convincing transgene expression in a large number of amygdala neurons. However AAV-0.4αCaMKII-GluN2A*, AAV-0.4αCaMKII-GluN2A, AAV-0.4αCaMKII-GluN2B, AAV-CMV-GluN2A, AAV-CMV-GluN2B, AAV-EFS-GluN2A, and AAV-TRE3G-GluN2A were not capable of conferring transgene expression and these results were consistent with our *in vitro* findings (Figure 6).

### Lentiviral plasmids confer spurious transgene expression *in vitro*, due to the presence of the RSV promoter at the 5′ end of the lentiviral genome

**Figure 7:**
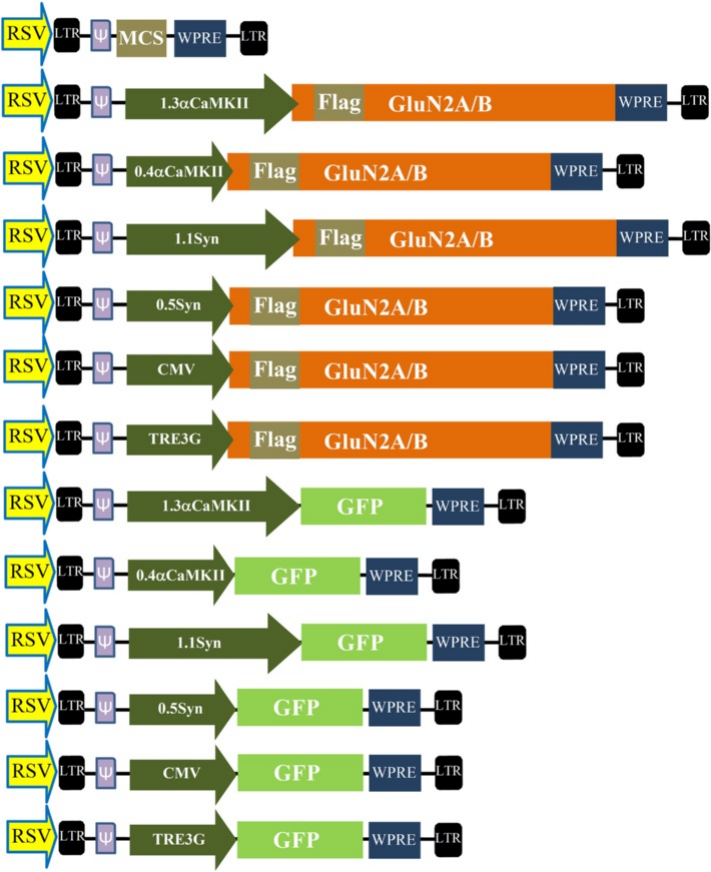
**Lentiviral vector maps.** Viral genome maps for pLenti7.3 vector backbones containing either the Flag-GluN2A/B or GFP transgenes. These transgenes are controlled by one of the following promoters as indicated: 1.3αCaMKII, 0.4αCaMKII, 1.1Synapsin, 0.5Synapsin, CMV, or TRE3G. The promoters are depicted as their approximate relative sizes to one another. GFP = green fluorescence protein, LTR = long terminal repeat, MCS = multiple cloning site, WPRE = woodchuck hepatitis post-transcriptional regulatory element, RSV = Rous Sarcoma virus promoter.

**Figure 8:**
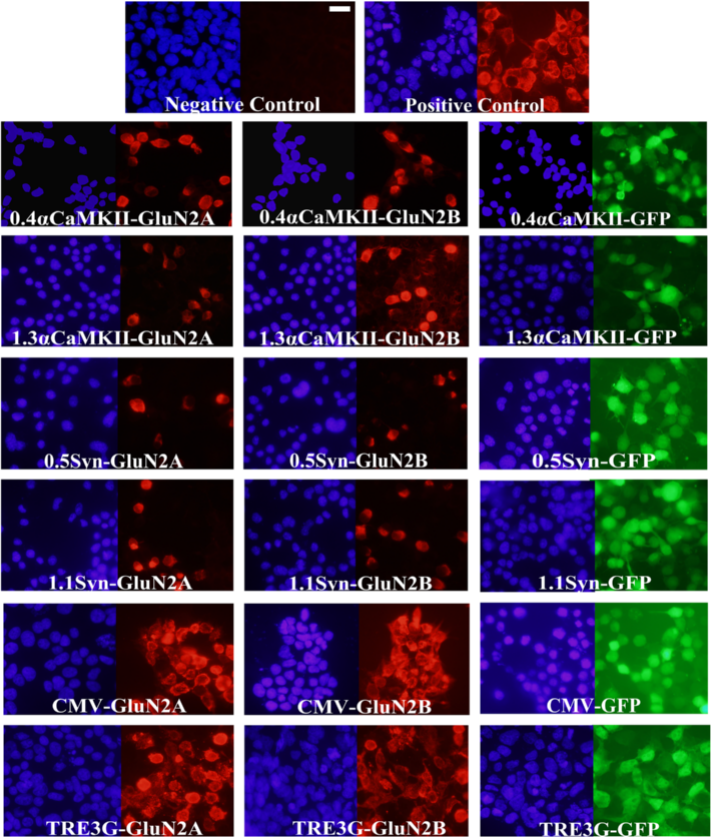
**GluN2 and GFP transgene expression** ***in vitro*** **from lentiviral vector plasmids.** Representative ICC images from transfections with lentiviral plasmids designed to express Flag-GluN2A/B or GFP transgenes utilizing one of the following promoters: 1.3αCaMKII, 0.4αCaMKII, 1.1Synapsin, 0.5Synapsin, CMV, or TRE3G. Plasmids containing transgenes controlled by the neuron specific, 0.4αCaMKII, 1.3αCaMKII, 0.5synapsin and 1.1synapsin promoters were transfected into N2A cells. Plasmids containing transgenes controlled by CMV and TRE3G promoters were transfected into 293FT cells. TRE3G promoter containing plasmids were co-transfected with the pTet-Off plasmid. Twenty four hours after transfection, native GFP expression was observed via fluorescence microscopy and Flag-GluN2 expression was observed via ICC and fluorescence microscopy. Images depict DAPI stained nuclei with the same fields viewed for GFP or Flag-GluN2 (Texas Red) transgene expression. All lentiviral plasmids were capable of conferring GluN2 or GFP expression as intended. Untransfected cells and cells transfected with pRK5-Flag-GluN2 plasmids were processed as negative and positive ICC controls respectively. (Scale bar = 20 μm).

**Figure 9:**
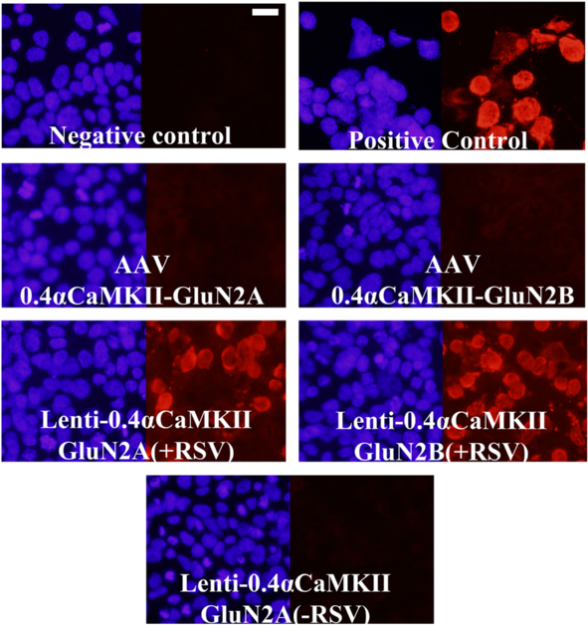
**Lentiviral plasmids confer spurious transgene expression** ***in vitro*** **, due to the presence of the RSV promoter at the 5′ end of the lentiviral genome.** In this experiment, AAV and lentiviral plasmids designed to express Flag-GluN2A/B from a 0.4αCaMKII promoter, and a lentiviral plasmid designed to express Flag-GluN2A from a 0.4αCaMKII promoter which had the RSV promoter deleted (−RSV), were transfected into 293FT cells. Twenty four hours after transfection Flag-GluN2 expression was observed via ICC and fluorescence microscopy. Images depict DAPI stained nuclei with the same fields viewed Flag-GluN2 (Texas Red) transgene expression. Similar to previous results, the AAV plasmids did not confer GluN2 expression and the lentiviral plasmids did confer GluN2 transgene expression. However the lentiviral plasmid without the RSV promoter did not confer 0.4αCaMKII-GluN2A transgene expression. Untransfected cells and cells transfected with pRK5-Flag-GluN2 plasmids were processed as negative and positive ICC controls respectively, (scale bar = 20 μm).

Because we were unable to develop AAV that could deliver and express the full length GluN2 transgenes effectively, we sought to develop a lentiviral vector that could deliver the GluN2 transgenes to neurons *in vivo* to confer ectopic expression of these transgenes. We started by modifying the pLenti.7.3 DEST vector (*Invitrogen*), by removing the emerald green fluorescent protein (emGFP) expression cassette and removing the DNA between the beginning of the CMV promoter to the beginning of the WPRE site and cloning in a multiple cloning site (MCS). This was performed to facilitate the cloning of transgenes of interest and to remove unessential DNA from the lentiviral genome to reduce the overall size of the viral genome as much as possible. The Flag-GluN2A, Flag-GluN2B, and GFP coding regions were cloned into this custom modified lenti vector and subsequently versions of these plasmids were generated to contain one of the following different promoters: 0.4αCaMKII, 1.3αCaMKII, 1.1Synapsin, 0.5Synapsin, CMV and TRE3G (Figure 7). These plasmids were transfected into cells lines, and the expression of the transgene was examined 24 hours post transfection as described above. The plasmids containing neuron specific promoters were transfected into N2A cells and the remaining plasmids were transfected into 293FT cells. Each of these plasmids exhibited transgene expression (Figure 8). This result was surprising since the neuron specific promoters were not capable of conferring detectable expression of full length GluN2 transgene expression when these transgenes were in the AAV vector plasmids. Additionally when the lentiviral plasmids that contained neuron specific promoters were transfected into 293FT cells, transgene expression was detected; however these neuron specific promoters do not confer appreciable transcriptional activation in this cell line. We hypothesized that the RSV promoter which is present at the beginning of the lentiviral genome plasmid, but not present in the lentivirus, could be responsible for this cryptic expression. To test this hypothesis we generated the lenti viral plasmid 0.4αCaMKII-GluN2A without the RSV promoter and transfected this plasmid into 293FT cells along with the unmodified version of the plasmid. Twenty four hours post transfection, the expression levels of GluNA were examined, and as expected, GluN2A transgene expression was not detected from cells transfected with the plasmid without the RSV promoter, indicating it was the RSV promoter that was responsible for this spurious transgene expression (Figure 9).

### Lentiviruses designed with TRE3G and CMV promoters conferred full length GluN2 expression *in vitro and in vivo*

Next we wanted to determine if the lentiviral plasmids designed to express GluN2A/B and GFP could be packaged into functional virus and if these viruses could be used to transduce cells *in vitro* and *in vivo* to induce ectopic transgene expression. Lentiviruses were produced using the following lentiviral vector plasmids: 0.4αCaMKII-GluN2A, 0.4αCaMKII-GluN2B, 1.3αCaMKII-GluN2A, 1.3αCaMKII-GluN2B, 1.1Syn-GluN2A, 1.1Syn-GluN2B, TRE3G-GluN2A, TRE3G-GluN2B, CMV-GluN2A, CMV-GluN2B, 0.4αCaMKII-GFP, 1.3αCaMKII-GFP, 1.1Syn-GFP, TRE3G-GFP, and CMV-GFP. The viruses that contained transgenes controlled by the CMV and TRE3G promoters were applied to 293FT cells *in vitro,* and 48 hours post application, the cells were examined for transgene expression as described above*.*

**Figure 10:**
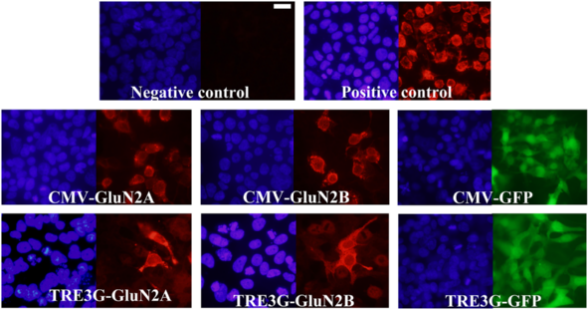
**GluN2 and GFP transgene expression** ***in vitro*** **mediated by lentivirus.** In this experiment, 293FT cells were transduced with lentiviruses designed to express GFP, Flag-GluN2A, or Flag-GluN2B from a CMV or a TRE3G promoter as indicated. TRE3G promoter containing viruses were transduced into cells that were also transfected with the pTet-Off plasmid. Forty eight hours after viral transduction, native GFP expression was observed via fluorescence microscopy and Flag-GluN2 expression was observed via ICC and fluorescence microscopy. Images depict DAPI stained nuclei with the same fields viewed for GFP or Flag-GluN2 (Texas Red) transgene expression. Non-transduced cells and cells transfected with pRK5-Flag-GluN2 plasmids were processed as negative and positive ICC controls respectively. These viruses were capable of conferring GluN2 or GFP transgene expression as intended.

**Figure 11:**
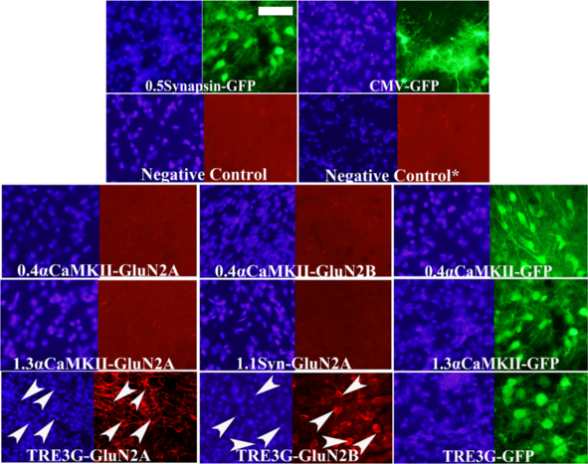
**GluN2 and GFP transgene expression** ***in vivo*** **mediated by lentivirus.** In this experiment lentiviruses designed to express Flag-GluN2A, Flag-GluN2B, or GFP under the control of 0.5Synapsin, CMV, 0.4αCaMKII, or 1.3αCaMKII promoters as indicated were infused into rat basal and lateral amygdala nuclei (BLA). Lentiviruses designed to express Flag-GluN2A, Flag-GluN2B, or GFP under the control of a TRE3G promoter were infused into αCaMKII-tTA transgenic mice. Ten days following viral infusion, coronal sections were prepared that contained the BLA and native GFP expression was observed via fluorescence microscopy and Flag-GluN2 expression was observed via immunohistochemistry, (IHC) and fluorescence microscopy. Images depict DAPI stained nuclei with the same fields viewed for GFP or Flag-GluN2 (Texas Red) transgene expression. Lentiviruses designed to express GFP from 0.5Synapsin, 0.4αCaMKII, 1.3αCaMKII, TRE3G promoters were capable of conferring GFP expression which was localized to neurons. Lentivirus designed to express GFP from a CMV promoter primarily conferred expression of GFP within glia cells. Lentiviruses designed to express Flag-GluN2A/B from either a 0.4αCaMKII or 1.3αCaMKII promoter were not capable of conferring GluN2 expression. Lentiviruses designed to express Flag-GluN2A/B from a TRE3G promoter were capable of conferring GluN2 expression as determined by IHC. Coronal sections from naïve controls were processed as a negative control for anti-Flag IHC (Negative control = coronal rat section and Negative control * = coronal mouse section), (scale bar = 50 μm).

The TRE3G-GluN2A, TRE3G-GluN2B, CMV-GluN2A, CMV-GluN2B, TRE3G-GFP, and CMV-GFP lentiviruses exhibited convincing and robust transgene expression (Figure 10). Because lentivirus transduces non-neuronal cells very efficiently *in vivo*, the CMV-GluN2A, CMV-GluN2B lentiviruses are not suitable for *in vivo* use since the transgenes will be expressed robustly in non-neuronal cells. This is unfortunate considering they do function as intended. However the TRE3G-GluN2A and TRE3G-GluN2B lentiviruses function as intended too and if these viruses are used within αCaMKII-tTA transgenic mice, the GluN2 transgenes will be specifically expressed in αCaMKII positive neurons. We have found that lentiviruses transduce 293FT cells very efficiently, however lentiviruses do not transduce N2A cells well and therefore we were not able to test the lentiviruses that contained transgenes controlled by the synapsin or αCaMKII promoters in cell culture – these were tested *in vivo*. The 0.4αCaMKII-GluN2A, 0.4αCaMKII-GluN2B, 1.3αCaMKII-GluN2A, 1.1Syn-GluN2A, TRE3G-GluN2A, TRE3G-GluN2B, 0.4αCaMKII-GFP, 1.3αCaMKII-GFP, 0.5Syn-GFP, TRE3G-GFP, and CMV-GFP lentiviruses were infused into the basolateral amygdala and 10 days later, the animals were sacrificed and the brains were sectioned in the coronal plane and the transgene expression was examined as above. All lentiviruses were infused into rats, except the viruses containing the TRE3G promoter. Viruses containing the TRE3G promoter were infused into αCaMKII-tTA mice [38]. Each of following lentiviruses exhibited transgene expression *in vivo*: TRE3G-GluN2A, TRE3G-GluN2B, 0.4αCaMKII-GFP, 1.3αCaMKII-GFP, 0.5Syn-GFP, TRE3G-GFP, and CMV-GFP (Figure 11). However none of the lentiviruses which contain neuronal specific promoters were capable of conferring *in vivo* expression of the full length GluN2 transgenes, which is consistent with our *in vitro* and *in vivo* AAV findings.

### Lentivirus designed with a 1.3 αCaMKII promoter and an intron conferred *in vivo* expression of full length GluN2A

**Figure 12:**
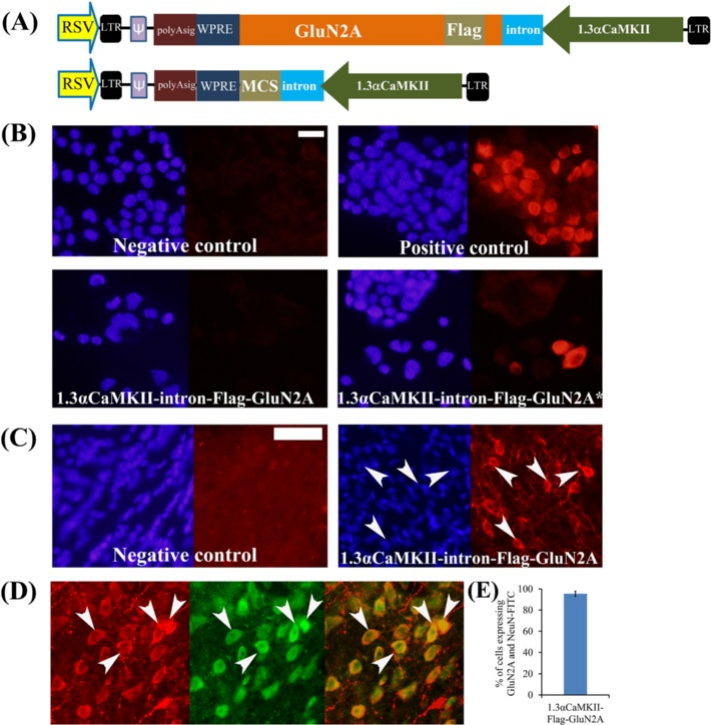
**Lentivirus designed with a 1.3** **αCaMKII promoter and an intron conferred** ***in vitro and in vivo*** **expression of full length GluN2A. (A)** Viral genome maps for pLenti7.3 vectors containing either the Flag-GluN2A coding region or a MCS with a 1.3αCaMKII promoter and intron. **(B)** A lentivirus plasmid designed to express Flag-GluN2A that included an intron from a 1.3αCaMKII promoter was transfected into N2A cells and 24 hours post transfection the cells were examined by ICC for Flag-GluN2 expression. This plasmid was not capable of conferring Flag-GluN2A expression. However when this plasmid was linearized, (Lenti-1.3αCaMKII + intron-GluN2A*) and then transfected as above to eliminate the RSV promoter from interfering with GluN2 expression, Flag-GluN2 expression was indeed detected. Non-transfected cells and cells transfected with the pRK5-Flag-GluN2A plasmid were processed as negative and positive ICC controls respectively (scale bar = 20 μm). **(C)** A lentivirus designed to express Flag-GluN2A that included an intron from a 1.3αCaMKII promoter was infused into the rat BLA. Ten days following viral infusion, coronal sections were prepared that contained the BLA and Flag-GluN2 expression was observed via IHC and fluorescence microscopy. Coronal sections from non-infused animals were processed as a negative control for Flag-IHC. **(D)** In this experiment, it was determined if the Flag-GluN2A expression from the 1.3αCaMKII-intron virus was predominantly restricted to neurons, by performing an IHC for Flag-GluN2A and the neuronal marker NeuN. Images depict Flag-GluN2A staining (Tx-red) with the same fields viewed for NeuN (FITC) staining and a merged image of these two images. This virus was capable of conferring GluN2A expression predominantly within neurons. Arrows point to a subset of NeuN positive cells that are also Flag-GluN2A positive **(E)** Quantification of data presented in (D), error bars represent the standard error of the mean, (scale bar = 50 μm).

**Figure 13:**
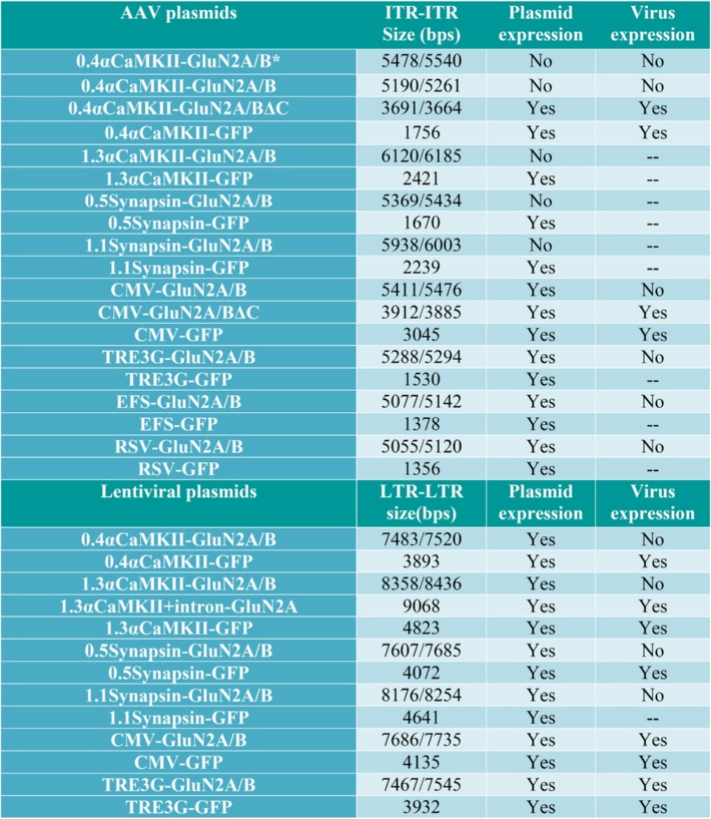
**Table summarizing results from the varying viral vectors produced in this study.**

In our final experiment we reasoned that including an intron within the GluN2 transgene might enhance expression of the transgene when used with a neuron specific promoter. However to our knowledge there are no commercially available lentiviral plasmids that contain introns and this is likely because lentiviruses are RNA viruses and these introns would be spliced out during viral production. To circumvent this issue, we developed a lentiviral vector that contained our transgene expression cassette in the reverse orientation and this expression cassette also contained an intron directly downstream of the 1.3αCaMKII promoter (Figure 12). By placing the transgene expression cassette in the reverse orientation, the transgene would not be expressed during viral production and therefore the intron would not be spliced out. We transfected this plasmid into N2A cells, and examined transgene expression 24 hours post transfection. However the GluN2A transgene expression was still undetectable. We reasoned that the opposing RSV promoter could be interfering with the 1.3αCaMKII-intron-GluN2A transgene expression. In order to confirm this, we linearized the plasmid to prevent the RSV promoter from interfering with the expression from the 1.3αCaMKII promoter, and transfected it into N2A cells. Cells transfected with the linearized plasmid exhibited positive GluN2A expression (Figure 12), thereby proving our hypothesis that the RSV promoter was interfering with the expression of the opposing transgene and that the inclusion of the intronic sequence led to enhanced expression of the transgene. Next we infused this virus into the rat basolateral amygdala and 10 days later, the animals were sacrificed and the brains were sectioned in the coronal plane and the transgene expression was examined as above (Figure 12). The 1.3 αCaMKII-Intron-GluN2A transgene was appropriately expressed in neurons *in vivo,* underscoring that combining the neuron specific promoter with an intron, in a lentivirus could confer expression of large and difficult to express transgene in a neuronal specific manner. We have also developed this lentiviral vector with a multiple cloning site so that any transgene can be easily cloned into this vector. Collectively the results from the viral vectors created for this study are summarized in Figure 13.

## Discussion

Creating viral vectors that can deliver large and or difficult to express transgenes to cells to direct robust ectopic expression is not trivial, however our findings coupled with the development of specific viral vectors should benefit others attempting to achieve this. We determined that viral packaging limit, transgene promoter and the presence of an intron within the transgene were all important factors that contributed to being able to successfully develop viral vectors designed to deliver and express GluN2 transgenes in a neuron specific manner. We initially developed lentiviruses designed to express the GluN2 transgenes with synapsin and αCaMKII promoters, but these viruses were never capable of conferring transgene expression *in vitro* or *in vivo* and it was unclear why. Because we could not initially develop lentivirus for this purpose, we began to work with AAV, despite knowing that AAVs packaging limit could create technical limitations. However working with AAV turned out to be fortuitous because it was during these experiments that we discovered that the synapsin and αCaMKII promoters were unable to confer expression of the GluN2 subunits, and that the transgene expression that was observed from lentiviral plasmids was in fact due to the lentiviral plasmid RSV promoter and not due to the neuron specific promoters. These findings were surprising considering that the synapsin and αCaMKII promoters are routinely used to direct neuron specific gene expression so we did not anticipate that they would not be capable of conferring detectable GluN2 expression. We were able to create functional lentiviruses that could express full length GluN2 transgenes using CMV and TRE3G promoters. And if the lentiviral transgene included an intron between the 1.3αCaMKII promoter and the GluN2A coding region, the 1.3αCaMKII promoter was capable of conferring neuron specific expression of GluN2A and functional viruses could be produced with this transgene. We also created this lentiviral plasmid with a multiple cloning site, so that any transgene can easily be cloned into it. The 1.3αCaMKII promoter-intron-MCS vector likely would be useful for anyone attempting to develop a lentivirus designed to express a difficult to express transgene that requires neuron specific expression.

Despite our best efforts, we were not able to create a functional adeno-associated virus that could confer full length GluN2 expression. These vectors were optimized so that they could be used for genes with large coding regions and this was accomplished by removing unnecessary DNA from the viral vector and by using short promoters and a ~75 bps 3′UTR that contained an SV40 based poly adenylation signal. However even with these modifications, the AAV-GluN2 viral genomes were all above 5 kb and this was due to the fact that the GluN2 coding regions are so large (~4.4 kb). It’s not necessarily surprising that the AAV-CMV-GluN2A, AAV-CMV-GluN2B, and AAV-TRE3G-GluN2A viruses were not capable of conferring GluN2 transgene expression, since these viruses were constructed with genomes that were much larger than the optimal genome size for AAV, (5411, 5476, 5228 bps respectively). However the AAV-EFS-GluN2A, and AAV-RSV-GluN2A possess genomes that were only marginally above the optimal packaging limit (5077 and 5055 bps respectively) and these also were not able to confer GluN2 transgene expression. Considering we have created functional AAV with genomes of similar sizes before (data not shown) and others have as well [13], we suspect that the AAV-EFS-GluN2A and AAV-RSV-GluN2A viruses were not capable of conferring expression of the GluN2 transgenes in part due to the less than optimal genome sizes and the fact that the EFS and RSV promoters do not confer robust expression of the GluN2 transgenes. Had we been able to find a promoter that was as short as or shorter than the RSV promoter (~225 bps), but also of similar strength to the CMV promoter, we suspect we would have been able to create a functional AAV that could confer GluN2 transgene expression. Regardless, these vectors have been optimized for transgenes with large coding regions and the existing GFP/GluN2 transgenes can easily be removed and any gene of interest can easily be cloned into these vectors – we suspect these viral plasmids can be useful for investigators interested in using AAV to express large transgenes.

There has been a desire to produce viruses that can deliver large transgenes for human gene therapy purposes and consequently there have been a number of studies examining this problem. For example numerous laboratories have been working on ways to develop AAV so it can deliver the large Cystic fibrosis transmembrane conductance regulator (CFTR) gene. The coding region of CFTR is ~4.6 kb. Previous studies have determined that the use of short promoters and short 3′ UTRs and the inclusion of an intron have facilitated production of AAV that can deliver and express CFTR [39,40] and these approaches were similar to what we have used to design viruses that can deliver GluN2 transgenes. However considering the CFTR transgene is capable of being expressed from significantly truncated promoters, it seems likely that the CFTR is not as difficult to express as the GluN2 transgenes. However more research would be required to determine this. Other approaches to develop AAV, so it can deliver large transgenes has involved a dual AAV system, where one AAV contains the promoter and some of the protein coding region of the transgene of interest and the other AAV contains the remaining protein coding region and the 3′ UTR containing a poly adenylation signal. These two AAVs also contain intron splice acceptor and donor signals and bridging sequences that are appropriately placed, so when these AAVs transduce cells, and the AAV genomes concatemerize, the complete transgene can be reconstituted, where the intron bridges the two viral genomes, resulting in an intact transgene. This approach holds promise for delivering large transgenes, but this approach also would result in reduced transgene expression compared to a single AAV that contained a complete transgene that was within the optimal AAV packaging limit [41]. Recently particular poly adenylation signal sequences have been identified that are short and therefore allow transgenes with larger coding regions to be packaged into AAV and these poly adenylation signal sequences were found to increase transgene expression [42]. In our study we included an intron within our lentiviral vector to facilitate GluN2A expression; however others have taken an alternative approach where they created hybrid promoters, by combining neuron specific promoters with portions of the CMV enhancer [43]. These hybrid promoters were capable of increasing expression, but in some cases these hybrid promoters exhibited less neuronal specificity.

We found that the neuron specific synapsin and αCaMKII, promoters were incapable of conferring detectable expression of full length GluN2 subunits and detectable expression could only be achieved from these promoters if the transgene included an intron or if the GluN2 subunit transgenes were truncated to only include the coding regions of the GluN2 transmembrane domains. It remains unclear if these transgenes were difficult to express simply due to their size or if there is something inherent to the mRNA regions that code for the C-terminal regions of the GluN2 protein or the C-terminal protein regions themselves that makes it difficult to express the full length GluN2 gene products. Recently AAV vectors were produced that were capable of delivering and expressing the Cas9 gene within neurons. The coding region of Cas9 is ~4.2 kb, which is slightly smaller than the coding regions for GluN2A and GluN2B. These vectors were designed with a truncated MeCP2 promoter and a short poly adenylation signal [44]. It remains to be determined how strong of a promoter this short MeCP2 promoter is and if it can confer neuronal specificity when used within lenti viruses. Considering Cas9 is very large and it appears to be adequately expressed from this short MeCP2 promoter and an EFS promoter (Addgene # 49535) [34], it seems likely that Cas9 is not necessarily difficult to express. We have found that Cas9 expresses well from an EFS promoter and truncated inducible promoters (data not shown).

Our findings underscore the importance of confirming that the viral plasmid transgene functions as intended before actually producing virus. While this is straightforward when using AAV plasmids, we found that with pLenti7.3 (*Invitrogen*) based lentiviral plasmids, it is not straightforward, since the viral 5′ RSV promoter conferred expression of our transgenes, essentially masking our ability to detect if the actual transgene promoter was capable of conferring expression of the transgene. All lentiviral genome plasmids contain a promoter at the 5′ end of the genome just prior to the right LTR, and this is so the viral genome can be transcribed in its entirety (R-LTR - L-LTR) when producing recombinant replication deficient lentivirus in cell culture. However this promoter is not present in the packaged viral RNA genome. We have not examined if other commercially available lentiviral plasmids confer expression of the transgenes within these plasmids from the 5′ promoter, but considering that all lentiviral plasmids possess a 5′ promoter and many utilize the RSV promoter, it is likely that other lentiviral plasmids exhibit this same phenomenon and this should be taken into account when confirming if the viral transgene is functioning correctly.

## Conclusion

In conclusion this study developed a number of viral vectors that could be of use to investigators in need of AAV and lenti vectors optimized for large or difficult to express transgenes. Additionally we have developed a number of viral vectors designed to express full length GluN2A and GluN2B, and GluN2A and B transgenes that possess deletions of their C-terminal cytoplasmic tails. These vectors could be very useful for investigators interested in examining the role of GluN2 in models of synaptic plasticity, learning and memory and psychiatric disease *in vitro* and *in vivo*. All viral vectors described in this study will be made available upon request and some of these will be made available through Addgene.

## Additional files

Additional file 1:
**Detailed description of how each viral plasmid was created for this study.**
Additional file 2:
**Viral genome sizes, and DNA oligonucleotides used for cloning purposes.**
Additional file 3:
**Promoter DNA sequences.**
Additional file 4:
**Additional relevant DNA sequences.**
Additional file 5:
**DNA vector sequences for all viral vectors produced for this study.**
