# Supplementary material for: The production of viral vectors designed to express large and difficult to express transgenes within neurons
Source: Mol Brain. 2015 Feb 24;8:12. doi: 10.1186/s13041-015-0100-7 (PMC4359567; doi:10.1186/s13041-015-0100-7)
Supplement: Additional file 1: — Detailed description of how each viral plasmid was created for this study. [file 13041_2015_100_MOESM1_ESM.docx]

**AAV vector design**

The 0.4 kb αCaMKII promoter was PCR amplified using the CaMKII FP MluI and CaMKII RP BglII primers and cloned into pAAV-Basic (Vector Biolabs) via the BglII and MluI sites. A 3’ UTR containing an SV40 based poly adenylation signal sequence was PCR amplified from pDsRedN1 (Clontech) using the Dsredutrfp (hindIII), dsredutrRP EcoRV primers and cloned into the HindIII and EcoRV sites and this vector is referred to as pAAV-Basic-0.4αCaMKII-MCS-3’UTR.

**Creation of pAAV vectors containing GluN2A**

The Flag tagged GluN2A open reading frame was PCR amplified from pRK5-Flag-GluN2A generously provided by Katherine Roche[[1](#_ENREF_1)] using (NR2A FP EcoR1, NR2A RP XhoI) primers and this PCR product was ligated into the EcoR1 and XhoI sites of pAAV-Basic-0.4αCaMKII-MCS-3’UTR to make pAAV-Basic-0.4αCaMKII-Flag-GluN2A. To reduce the size of the AAV genome size, from 5478 bps to 5190 bps, a different AAV vector was used referred to here as pAAV. This vector was systematically gutted to remove the intervening DNA between the ITRs and the new promoter, ORF, and 3’UTR were added. First the 0.4 kb αCaMKII promoter was PCR amplified using (CamkII FP MluI, CamkII RP EcoR1) primers and this PCR product was ligated into pAAV via the MluI and EcoR1 sites to create pAAV-0.4αCaMKII. Then a short 3’UTR was created by annealing the following oligonucleotides (3’UTR TOP, 3’ UTR Bot) and this was ligated into the XhoI and RsrII sites of pAAV-0.4αCaMKII to create pAAV-0.4αCaMKII-3’UTR. Finally the GluN2A ORF was removed from pAAV-Basic-0.4αCaMKII-Flag-GluN2A via the EcoR1 and XhoI sites and this DNA fragment was ligated into pAAV-0.4αCaMKII-3’UTR to create pAAV-0.4αCaMKII-Flag-GluN2A. The TRE3G, 1.3αCaMKII, 1.1 Synapsin, 0.5 synapsin, EFS and RSV promoters were PCR amplified using the following DNA primers (TRE3g FP 2 MluI, TRE3g RP EcoRI, CamkII 1.2kb MluI FP New, CamKII 1.2kb EcoR1 RP, Syn1.1FPMluI, Syn .5FPMluI, Syn1.1RPEcoR1, EFS FP MluI, EFS RP EcoR1, RSV FP MluI, and RSV RP EcoR1) and these gene products were ligated into the MluI and EcoR1 sites of pAAV-0.4αCaMKII-Flag-GluN2A to create, pAAV-TRE3G-Flag-GluN2A, pAAV-1.3αCaMKII-Flag-GluN2A, pAAV-1.1Syn-Flag-GluN2A, pAAV-0.5Syn-Flag-GluN2A, pAAV-EFS-Flag-GluN2A, pAAV-RSV-Flag-GluN2A respectively.

To create GluN2A expression vector that expressed a GluN2A without its cytoplasmic tail, pAAV-Basic-0.4αCaMKII-Flag-GluN2A was digested with SapI and XhoI and the annealed oligos (Nr2A STOP Top, Nr2A STOP Bot) were ligated into the SapI and XhoI sites of pAAV-Basic-0.4αCaMKII-Flag-GluN2A to create pAAV-Basic-0.4αCaMKII-Flag-GluN2A-CtermΔ.This plasmid is designed to express a truncated GluN2A with a stop codon that occurs directly after amino acid 871 (stop codon at base 2613). Subsequently the CMV promoter was PCR amplified using (CMV P FP MluI, CMV P RP R1) and ligated into the MluI and EcoR1 sites of pAAV-Basic-0.4αCaMKII-Flag-GluN2A-CtermΔ to create pAAV-Basic-CMV-Flag-GluN2A-CtermΔ

**Creation of pAAV vectors containing GFP**

GFP was PCR amplified using (GFP FP EcoR1, GFP RP XhoI) DNA primers and this PCR product was ligated into the EcoR1 and XhoI sites of pAAV-0.5Syn-Flag-GluN2A, to create pAAV-0.5Syn-GFP. The TRE3G, 1.3αCaMKII, 1.1 Synapsin, and RSV promoters were PCR amplified using the following DNA primers (TRE3g FP 2 MluI, TRE3g RP EcoRI, CamkII 1.2kb MluI FP New, CamKII 1.2kb EcoR1 RP, Syn1.1FPMluI, Syn1.1RPEcoR1, RSV FP MluI, and RSV RP EcoR1) and these gene products were ligated into the MluI and EcoR1 sites of pAAV-0.5Syn-GFP to create pAAV-TRE3G-GFP, pAAV-1.3αCaMKII-GFP, pAAV-1.1Syn-GFP, and pAAV-RSV-GFP respectively. GFP was PCR amplified using (GFP FP EcoR1, GFP RP XhoI) DNA primers and this PCR product was ligated into the EcoR1 and XhoI sites of pAAV-EFS-Flag-GluN2A, to create pAAV-EFS-GFP. GFP was PCR amplified using (GFP FP BglII, GFP RP HindIII) DNA primers and this PCR product was ligated into the BglII and HindIII sites of pAAV-Basic-0.4αCaMKII-MCS-3’UTR to create pAAV-Basic-0.4αCaMKII-GFP. The pAAV-shRNA expression cassette vector ^[^[^2^](#_ENREF_2)^]^,was digested with AscI and XbaI, the DNA ends were blunted and religated to remove the shRNA expression cassette, to create pAAV-CMV-GFP.

**Creation of pAAV vectors containing GluN2B**

The Flag tagged GluN2B open reading frame was PCR amplified from pRK5-Flag-GluN2B generously provided by Katherine Roche ^[^[^1^](#_ENREF_1)^]^using (NR2B FP BamHI, NR2B RP EcoRI), primers and this PCR product was ligated into the BamHI and EcoRI sites of pAAV-Basic-0.4αCaMKII-MCS-3’UTR to make pAAV-Basic-0.4αCaMKII-Flag-GluN2B. To reduce the size of the AAV genome size, from 5540 bps to 5261 bps, the 0.4CaMKII promoter and GluN2B ORF were removed from pAAV-Basic-0.4αCaMKII-Flag-GluN2B via the MluI and EcoR1 sites and ligated into pAAV-0.4αCaMKII-3’UTR via the MluI and EcoRI sites. Subsequently this vector was digested with EcoRI and XhoI, the DNA ends were blunted and ligated to remove intervening DNA between these sites to create pAAV-0.4αCaMKII-Flag-GluN2B.

The TRE3G, 1.3αCaMKII, 1.1 Synapsin, 0.5 synapsin, EFS, RSV and CMV promoters were PCR amplified using the following DNA primers (TRE3g FP 2 MluI, TRE3g RP BamHI, CamkII 1.2kb MluI FP New, CamKII1.2kb BamHII RP P, Syn1.1FPMluI, Syn .5FPMluI, Syn1.1RPBamHI, EFS FP MluI, EFS RP BamHI, RSV FP MluI, RSV RP BamHI, CMV P FP MluI, CMV P RP BamHI) and these gene products were ligated into the MluI and BamHI sites of pAAV-0.4αCaMKII-Flag-GluN2B to create, pAAV-TRE3G-Flag-GluN2B, pAAV-1.3αCaMKII-Flag-GluN2B, pAAV-1.1Syn-Flag-GluN2B, pAAV-0.5Syn-Flag-GluN2B, pAAV-EFS-Flag-GluN2B, pAAV-RSV-Flag-GluN2B and pAAV-CMV-Flag-GluN2B respectively.

To create a GluN2B expression vector that expressed GluN2B without its cytoplasmic tail, the GluN2B ORF excluding the cytoplasmic tail region was PCR amplified using (NR2B FP BamHI , Nr2Bctermdeletion RP) primers and the PCR product was ligated into pAAV-Basic-0.4αCaMKII-MCS-3’UTR vector via the BamHI and EcoR1 sites to create pAAV-Basic-0.4αCaMKII-Flag-GluN2B-CtermΔ. This plasmid is designed to express a truncated GluN2B with a stop codon that occurs directly after amino acid 867 (stop at base 2601). Subsequently the CMV promoter was PCR amplified using (CMV P FP MluI, CMV P RP BamHI) and ligated into the MluI and BamHI sites of pAAV-Basic-0.4αCaMKII-Flag-GluN2B-CtermΔ to create pAAV-Basic-CMV-Flag-GluN2B-CtermΔ

**Lenti Virus Vector Design**

The lenti viruses created for this study used the lenti viral genome Gateway recombination based cloning vector pLenti7.3/V5-DEST plasmid (Invitrogen). This vector was subsequently modified by removing the emGFP expression cassette via the Acc65I sites and is referred to as pLenti7.3(-emGFP). The removal of this expression cassette reduced the viral genome by 1093 bases. To convert pLenti7.3(-emGFP) vector from a Gateway recombination based cloning vector to a standard ligation dependent multiple cloning site containing vector, the vector was digested with ClaI and MluI and the oligos, (LentiMCS TOP, and LentiMCS Bot) were annealed and ligated to the ClaI and MluI sites of this vector to create pLentiMCS(-emGFP).

**Creation of Lenti viral plasmids containing GluN2A**

pLenti(MCS)-0.4αCaMKII-FlagGluN2A was created by excising the 0.4αCaMKII promoter and FlagGluN2A transgene from pAAV-Basic-0.4αCamkII-FlagGluN2A via the MluI and XhoI restriction enzyme sites and cloning it into the MluI and XhoI restriction enzymes sites within pLentiMCS(-emGFP) to create pLentiMCS(-emGFP)0.4αCaMKII-FlagGluN2A. To create pLentiMCS(-emGFP)CMV-FlagGluN2A, pLentiMCS(-emGFP)0.4αCaMKII-FlagGluN2A was digested with MluI and BlpI and the MluI-BlpI fragment from pAAV-CMV-FlagGluN2A containing the CMV promoter and portion of GluN2A was ligated into the into MluI and BlpI sites of pLentiMCS(-emGFP)0.4αCaMKII-FlagGluN2A. To remove an EcoR1 site that is after the stop codon of the GluN2A transgene within pLentiMCS(-emGFP)0.4αCaMKII-FlagGluN2A, the vector was digested with XhoI and AgeI and religated, to create pLentiMCS(-emGFP)0.4αCaMKII-FlagGluN2A(mod). The TRE3G, 1.3 CaMKII, 1.1 Synapsin, and 0.5 synapsin promoters were PCR amplified using the following DNA primers (TRE3g FP 2 MluI, TRE3g RP EcoRI, CamkII 1.2kb MluI FP New, CamKII 1.2kb EcoR1 RP, Syn1.1FPMluI, Syn .5FPMluI, Syn1.1RPEcoR1) and these PCR products were ligated into pLentiMCS(-emGFP)0.4αCaMKII-FlagGluN2A(mod) via the MluI and EcoRI sites to create, pLentiMCS(-emGFP)TRE3G-FlagGluN2A(mod), pLentiMCS(-emGFP)1.3CaMKII-FlagGluN2A(mod), pLentiMCS(-emGFP)1.1Syn-FlagGluN2A(mod), and pLenti(MCS(-emGFP)0.5Syn-FlagGluN2A(mod) respectively.

To create pLenti(MCS)-0.4αCaMKII-FlagGluN2A(-RSV), a double digest with SphI and KasI enzymes was performed on pLenti(MCS)-0.4αCaMKII-FlagGluN2A to excise a portion of the RSV promoter and the ends of the vector were blunted and the vector was religated. To create pLentiMCS(-emGFP)1.3αCaMKII-Intron-FlagGluN2A(mod)-WPRE-polyASig, the 1.3αCaMKII promoter with an intron was PCR amplified using primers (CamkII 1.2KB FP Acc65I, camKII pro w Intron RP) from pAAV-CaMKII-RFP Addgene # 22908[[3](#_ENREF_3)]) and this fragment was cloned into pLentiMCS(-emGFP) via the Acc65I and EcoRI sites to create pLentiMCS(-emGFP)1.3αCaMKII-Intron. Subsequently the Flag-GluN2A was removed from pAAV-Basic-0.4αCaMKII-Flag-GluN2A via the EcoR1 and XhoI sites and inserted into pLentiMCS(-emGFP)1.3αCaMKII-Intron via these same sites. This vector then had a 3’UTR containing an SV40 based poly A signal sequence inserted, by annealing the following oligonucleotides (3’UTR TOP XhoI P, 3’UTR Bot XhoI P) and ligated them into pLentiMCS(-emGFP)1.3αCaMKII-Intron-Flag-GluN2A via the XhoI sites. Finally a DNA seqeuence containing a WPRE sequence was PCR amplified from pLenti7.3/V5-DEST with the PCR primers (WPRE FP XhoI, WPRE RP XhoI) and ligated into the XhoI site in pLentiMCS(-emGFP)1.3αCaMKII-Intron-Flag-GluN2A-PolyAsig, to make pLentiMCS(-emGFP)1.3αCaMKII-Intron-Flag-GluN2A-WPRE-PolyAsig. The pLentiMCS(-emGFP)1.3αCaMKII-Intron-MCS-WPRE-PolyAsig vector was created by removing the GluN2A transgene via the EcoR1 and SalI sites from pLentiMCS(-emGFP)1.3αCaMKII-Intron-Flag-GluN2A-WPRE-PolyAsig, and replacing it with the annealed oligos (Lenti camKII Intron MCS TOP, Lenti camKII Intron MCS Bot).

**Creation of Lenti viral plasmids containing GFP**

The open reading frame for GFP was PCR amplified with the PCR primers (GFP attB FP, GFP attB RP) and inserted into pDONR221(Invitrogen) via a BP clonase reaction to create pDONR221-GFP. GFP was subsequently transferred to pLenti7.3(-emGFP) via LR clonase reaction to create pLenti7.3(-emGFP)-GFP. Subsequently the CMV promoter was removed from pLenti7.3(-emGFP)-GFP via the ClaI and SpeI sites and the 0.4CamKII, 1.3αCamKII, 1.1 Synapsin, and 0.5synapsin promoters were inserted into these sites. These promoters were PCR amplified using the following primers (CamkII FP claI, CamkII RP speI, mCamkII FP 1.2 claI, Syn1.1FPClaI, Syn1.1RPSpeI, Syn5.5FPClaI). The 0.4CamKII promoter was PCR amplified from rat genomic DNA. The 1.3αCamKII promoter was PCR amplified from pAAV-CaMKII-RFP Addgene # 22908 [[3](#_ENREF_3)]. The Synapsin promoters were PCR amplified from the L26 plasmid generously provided by Pavel Osten [[4](#_ENREF_4)].

**Creation of Lenti viral plasmids containing GluN2B**

pLenti(MCS)-0.4αCaMKII-FlagGluN2B was created by excising the 0.4αCaMKII promoter and FlagGluN2B transgene from pAAV-Basic-0.4αCamkII-FlagGluN2B via the MluI and EcoR1 restriction enzyme sites and cloning it into the MluI and EcoR1 restriction enzymes sites within pLentiMCS(-emGFP) to create pLentiMCS(-emGFP)0.4αCaMKII-FlagGluN2B. To create pLentiMCS(-emGFP)CMV-FlagGluN2B, pLentiMCS(-emGFP)0.4αCaMKII-FlagGluN2B was digested with MluI and XbaI and the MluI-XbaI fragment from pAAV-CMV-FlagGluN2B containing the CMV promoter and portion of GluN2B was ligated into the into MluI and XbaI sites of pLentiMCS(-emGFP)0.4αCaMKII-FlagGluN2B. To remove a BamHI site that is after the stop codon of the GluN2B transgene within pLentiMCS(-emGFP)0.4αCaMKII-FlagGluN2B, the vector was digested with EcoR1 and partially digested with BamHI and religated to destroy the BamHI, to create pLentiMCS(-emGFP)0.4CaMKII-FlagGluN2B(mod). The TRE3G, 1.3αCaMKII, 1.1 Synapsin, and 0.5 synapsin promoters were PCR amplified using the following DNA primers (TRE3g FP 2 MluI, TRE3g RP BamHI, CamkII 1.2kb MluI FP New, CamKII1.2kb BamHII RP, Syn1.1FPMluI, Syn .5FPMluI, Syn1.1RPBamHI) and these PCR products were ligated into pLentiMCS(-emGFP)0.4αCaMKII-FlagGluN2B(mod) via the MluI and BamHI sites to create, pLentiMCS(-emGFP)TRE3G-FlagGluN2B(mod), pLentiMCS(-emGFP)1.3αCaMKII-FlagGluN2B(mod), pLentiMCS(-emGFP)1.1Syn-FlagGluN2B(mod), and pLentiMCS(-emGFP)0.5Syn-FlagGluN2B(mod) respectively.

1. Lavezzari G, McCallum J, Dewey CM, Roche KW: **Subunit-specific regulation of NMDA receptor endocytosis**. *The Journal of neuroscience : the official journal of the Society for Neuroscience* 2004, **24**(28):6383-6391.

2. Hommel JD, Sears RM, Georgescu D, Simmons DL, DiLeone RJ: **Local gene knockdown in the brain using viral-mediated RNA interference**. *Nature medicine* 2003, **9**(12):1539-1544.

3. Nathanson JL, Yanagawa Y, Obata K, Callaway EM: **Preferential labeling of inhibitory and excitatory cortical neurons by endogenous tropism of adeno-associated virus and lentivirus vectors**. *Neuroscience* 2009, **161**(2):441-450.

4. Dittgen T, Nimmerjahn A, Komai S, Licznerski P, Waters J, Margrie TW, Helmchen F, Denk W, Brecht M, Osten P: **Lentivirus-based genetic manipulations of cortical neurons and their optical and electrophysiological monitoring in vivo**. *Proceedings of the National Academy of Sciences of the United States of America* 2004, **101**(52):18206-18211.
