# Supplementary material for: The production of viral vectors designed to express large and difficult to express transgenes within neurons
Source: Mol Brain. 2015 Feb 24;8:12. doi: 10.1186/s13041-015-0100-7 (PMC4359567; doi:10.1186/s13041-015-0100-7)
Supplement: Additional file 3: — Promoter DNA sequences. [file 13041_2015_100_MOESM3_ESM.docx]

>EFS promoter 251 bases.

tcgagtggctccggtgcccgtcagtgggcagagcgcacatcgcccacagtccccgagaag

ttggggggaggggtcggcaattgaaccggtgcctagagaaggtggcgcggggtaaactgg

gaaagtgatgtcgtgtactggctccgcctttttcccgagggtgggggagaaccgtatata

agtgcagtagtcgccgtgaacgttctttttcgcaacgggtttgccgccagaacacaggtg

tcgtgacgcgg

>0.4kb Rat Alpha CaMKII promoter 364 bases.

acttgtggactaagtttgttcacatccccttctccaaccccctcagtacatcaccctggg

agaacaaggtccacttgcttctgggcccacacagtcctgcagtattgtgtatataaggcc

agggcaacggaggagcaggttttgaagtgaaaggcaggcaggtgttggggaggcagttac

cggggcaacgggaacagggcgtttcggaggtggttgccatggggacctggatgctgacga

aggctcgcgaggctgtgagcagccacagtgccctgctcagaagccccaagctcgtcaatc

aagctggttctccatttgcactcaggagcacgggcaggcgagtggcccctagttctggga

gcag

>1.3kb mouse Alpha CaMKII promoter 1294 bases.

tcgtaacattatggccttaggtcacttcatctccatggggttcttcttctgattttctag

aaaatgagatgggggtgcagagagcttcctcagtgacctgcccagggtcacatcagaaat

gtcagagctagaacttgaactcagattactaatcttaaattccatgccttgggggcatgc

aagtacgatatacagaaggagtgaactcattagggcagatgaccaatgagtttaggaaag

aagagtccagggcagggtacatctacaccacccgcccagccctgggtgagtccagccacg

ttcacctcattatagttgcctctctccagtcctaccttgacgggaagcacaagcagaaac

tgggacaggagccccaggagaccaaatcttcatggtccctctgggaggatgggtggggag

agctgtggcagaggcctcaggaggggccctgctgctcagtggtgacagataggggtgaga

aagcagacagagtcattccgtcagcattctgggtctgtttggtacttcttctcacgctaa

ggtggcggtgtgatatgcacaatggctaaaaagcagggagagctggaaagaaacaaggac

agagacagaggccaagtcaaccagaccaattcccagaggaagcaaagaaaccattacaga

gactacaagggggaagggaaggagagatgaattagcttcccctgtaaaccttagaaccca

gctgttgccagggcaacggggcaatacctgtctcttcagaggagatgaagttgccagggt

aactacatcctgtctttctcaaggaccatcccagaatgtggcacccactagccgttacca

tagcaactgcctctttgccccacttaatcccatcccgtctgttaaaagggccctatagtt

ggaggtgggggaggtaggaagagcgatgatcacttgtggactaagtttgttcgcatcccc

ttctccaaccccctcagtacatcaccctgggggaacagggtccacttgctcctgggccca

cacagtcctgcagtattgtgtatataaggccagggcaaagaggagcaggttttaaagtga

aaggcaggcaggtgttggggaggcagttaccggggcaacgggaacagggcgtttcggagg

tggttgccatggggacctggatgctgacgaaggctcgcgaggctgtgagcagccacagtg

ccctgctcagaagccccaagctcgtcagtcaagccggttctccgtttgcactcaggagca

cgggcaggcgaGTGGCCCCTAGTTCTGGGAGCAG

>CMV promoter 585 bases.

ggagttccgcgttacataacttacggtaaatggcccgcctggctgaccgcccaacgaccc

ccgcccattgacgtcaataatgacgtatgttcccatagtaacgccaatagggactttcca

ttgacgtcaatgggtggagtatttacggtaaactgcccacttggcagtacatcaagtgta

tcatatgccaagtacgccccctattgacgtcaatgacggtaaatggcccgcctggcatta

tgcccagtacatgaccttatgggactttcctacttggcagtacatctacgtattagtcat

cgctattaccatggtgatgcggttttggcagtacatcaatgggcgtggatagcggtttga

ctcacggggatttccaagtctccaccccattgacgtcaatgggagtttgttttggcacca

aaatcaacgggactttccaaaatgtcgtaacaactccgccccattgacgcaaatgggcgg

taggcgtgtacggtgggaggtctatataagcagagctcgtttagtgaaccgtcagatcgc

ctggagacgccatccacgctgttttgacctccatagaagacaccg

>TRE3G promoter 403 bases.

atcacgagactagcctcgagagttggctttactccctatcagtgatagagaacgtatgaa

gagtttactccctatcagtgatagagaacgtatgcagactttactccctatcagtgatag

agaacgtataaggagtttactccctatcagtgatagagaacgtatgaccagtttactccc

tatcagtgatagagaacgtatctacagtttactccctatcagtgatagagaacgtatatc

cagtttactccctatcagtgatagagaacgtataagctttaggcgtgtacggtgggcgcc

tataaaagcagagctcgtttagtgaaccgtcagatcgcctggagcaattccacaacactt

ttgtcttataccaactttccgtaccacttcctaccctcgtaaa

>RSV promoter 229 bases.

aatgtagtcttatgcaatactcttgtagtcttgcaacatggtaacgatgagttagcaaca

tgccttacaaggagagaaaaagcaccgtgcatgccgattggtggaagtaaggtggtacga

tcgtgccttattaggaaggcaacagacgggtctgacatggattggacgaaccactgaatt

gccgcattgcagagatattgtatttaagtgcctagctcgatacataaac

>1.1 Rat Synapsin promoter 1112 bases.

gggttttggctacgtccagagcagaggaatgagggcatgtagactaaatatgttcgtgtg

gaagaggctgaatacacatcagagttactgctgcaggaaatgcttctgcattgcataccc

agagtttccttgctcatctgagagcatgtgttttttccagatgtgtgtacttgtgtgaga

ttctctgggtgtgtgtcaatgtgttgcctgaacgtgcattgctcaatatgctcatgtgtg

ttaccctgggcttgtacatctacatatatacctggatgcccgtgtgttctgtgatgtaca

tataccctgtgtcattccttgtttttctatttgtgttattccatgtgttccttcaggctc

tcactacccaagtgtccacctccgcctgtctggtgatgtttacgctaccccgtgctcttt

tctttgcctgacagtgttgtcgtggaagacatctcgccaggaacactgcagtaaggagaa

tttctagttttatgttcccctccgagtatgcttctatcccgaccctcaaccccaaaatgc

cttcagaggtgaaaatcaacactggaaacacaagtatctgggaagggtaacaatgcaagt

tagcctgaggatttaggaggaggctgaaaaacagagtaggagccttactacgggtccaga

ccctacggacaagaacccccactcccactccccaaattgcgcattccctcccccatcaga

gggggaggggaagaggatgcagcgcggcgcggcgcgtgcgcactgtcggatttagtaccg

cggacagagccttcgcccccgctgccggcgcgcgccaccacctccccagcaccaaaggcg

ggctgacgtcactctccagccctccccaaactcccctacctcaccgccttggtcgcgtcc

gtgcagcggtgagtccagtcgggccgcaccacaagaggtgcaagataggggggtgcaggc

gcgaccatacgctctgcggcggcagagcctcagcgctgcctcagtctgcagcgggcagca

gaggagtcgcgtcgtgccagagagcgccgccgtgctcctgagccccttgcgctccgcccc

cgcggcccaccgacccactgccccttggatcc

>0.5 Rat Synapsin promoter 543 bases.

acaagtatctgggaagggtaacaatgcaagttagcctgaggatttaggaggaggctgaaa

aacagagtaggagccttactacgggtccagaccctacggacaagaacccccactcccact

ccccaaattgcgcattccctcccccatcagagggggaggggaagaggatgcagcgcggcg

cggcgcgtgcgcactgtcggatttagtaccgcggacagagccttcgcccccgctgccggc

gcgcgccaccacctccccagcaccaaaggcgggctgacgtcactctccagccctccccaa

actcccctacctcaccgccttggtcgcgtccgtgcagcggtgagtccagtcgggccgcac

cacaagaggtgcaagataggggggtgcaggcgcgaccatacgctctgcggcggcagagcc

tcagcgctgcctcagtctgcagcgggcagcagaggagtcgcgtcgtgccagagagcgccg

ccgtgctcctgagccccttgcgctccgcccccgcggcccaccgacccactgccccttgga

tcc
