# Supplementary material for: The production of viral vectors designed to express large and difficult to express transgenes within neurons
Source: Mol Brain. 2015 Feb 24;8:12. doi: 10.1186/s13041-015-0100-7 (PMC4359567; doi:10.1186/s13041-015-0100-7)
Supplement: Additional file 4: — Additional relevant DNA sequences. [file 13041_2015_100_MOESM4_ESM.docx]

>L-ITR 141 bases.

CCTGCAGGCAGCTGCGCGCTCGCTCGCTCACTGAGGCCGCCCGGGCAAAGCCCGGGCGTC

GGGCGACCTTTGGTCGCCCGGCCTCAGTGAGCGAGCGAGCGCGCAGAGAGGGAGTGGCCA

ACTCCATCACTAGGGGTTCCT

>R-ITR 141 bases.

AGGAACCCCTAGTGATGGAGTTGGCCACTCCCTCTCTGCGCGCTCGCTCGCTCACTGAGG

CCGGGCGACCAAAGGTCGCCCGACGCCCGGGCTTTGCCCGGGCGGCCTCAGTGAGCGAGC

GAGCGCGCAGCTGCCTGCAGG

>SV40 based poly A signal Sequence from pDsRedN1 219 bases.

cacatttgtagaggttttacttgctttaaaaaacctcccacacctccccctgaacctgaa

acataaaatgaatgcaattgttgttgttaacttgtttattgcagcttataatggttacaa

ataaagcaatagcatcacaaatttcacaaataaagcatttttttcactgcattctagttg

tggtttgtccaaactcatcaatgtatcttaaggcgtaaa

>shortened SV40 based poly A signal Sequence from pDsRedN1 76 bases.

Gtttattgcagcttataatggttacaaataaagcaatagcatcacaaatttcacaaataa

Agcatttttttcactg

>eGFP 720 bases.

atggtgagcaagggcgaggagctgttcaccggggtggtgcccatcctggtcgagctggac

ggcgacgtaaacggccacaagttcagcgtgtccggcgagggcgagggcgatgccacctac

ggcaagctgaccctgaagttcatctgcaccaccggcaagctgcccgtgccctggcccacc

ctcgtgaccaccctgacctacggcgtgcagtgcttcagccgctaccccgaccacatgaag

cagcacgacttcttcaagtccgccatgcccgaaggctacgtccaggagcgcaccatcttc

ttcaaggacgacggcaactacaagacccgcgccgaggtgaagttcgagggcgacaccctg

gtgaaccgcatcgagctgaagggcatcgacttcaaggaggacggcaacatcctggggcac

aagctggagtacaactacaacagccacaacgtctatatcatggccgacaagcagaagaac

ggcatcaaggtgaacttcaagatccgccacaacatcgaggacggcagcgtgcagctcgcc

gaccactaccagcagaacacccccatcggcgacggccccgtgctgctgcccgacaaccac

tacctgagcacccagtccgccctgagcaaagaccccaacgagaagcgcgatcacatggtc

ctgctggagttcgtgaccgccgccgggatcactctcggcatggacgagctgtacaagtaa
